# A Novel Subset of CD95^+^ Pro-Inflammatory Macrophages Overcome miR155 Deficiency and May Serve as a Switch From Metabolically Healthy Obesity to Metabolically Unhealthy Obesity

**DOI:** 10.3389/fimmu.2020.619951

**Published:** 2021-01-07

**Authors:** Candice Johnson, Charles Drummer IV, Huimin Shan, Ying Shao, Yu Sun, Yifan Lu, Fatma Saaoud, Keman Xu, Gayani Nanayakkara, Pu Fang, Zsolt Bagi, Xiaohua Jiang, Eric T. Choi, Hong Wang, Xiaofeng Yang

**Affiliations:** ^1^ Cardiovascular Research Center, Lewis Katz School of Medicine at Temple University, Philadelphia, PA, United States; ^2^ Metabolic Disease Research, Lewis Katz School of Medicine at Temple University, Philadelphia, PA, United States; ^3^ Vascular Biology Center, Augusta University, Augusta, GA, United States; ^4^ Division of Vascular and Endovascular Surgery, Department of Surgery, Lewis Katz School of Medicine at Temple University, Philadelphia, PA, United States; ^5^ Departments of Pharmacology, Microbiology and Immunology, Lewis Katz School of Medicine at Temple University, Philadelphia, PA, United States; ^6^ Centers for Inflammation, Translational and Clinical Lung Research, Lewis Katz School of Medicine at Temple University, Philadelphia, PA, United States

**Keywords:** Macrophage, CD95 (Fas), atherosclerosis, metabolic disease, obesity, metabolic, miR-155, adipose tissue

## Abstract

Metabolically healthy obesity (MHO) accounts for roughly 35% of all obese patients. There is no clear consensus that has been reached on whether MHO is a stable condition or merely a transitory period between metabolically healthy lean and metabolically unhealthy obesity (MUO). Additionally, the mechanisms underlying MHO and any transition to MUO are not clear. Macrophages are the most common immune cells in adipose tissues and have a significant presence in atherosclerosis. Fas (or CD95), which is highly expressed on macrophages, is classically recognized as a pro-apoptotic cell surface receptor. However, Fas also plays a significant role as a pro-inflammatory molecule. Previously, we established a mouse model (ApoE^-/-^/miR155^-/-^; DKO mouse) of MHO, based on the criteria of not having metabolic syndrome (MetS) and insulin resistance (IR). In our current study, we hypothesized that MHO is a transition phase toward MUO, and that inflammation driven by our newly classified CD95^+^CD86^-^ macrophages is a novel mechanism for this transition. We found that, with extended (24 weeks) high-fat diet feeding (HFD), MHO mice became MUO, shown by increased atherosclerosis. Mechanistically, we found the following: *1)* at the MHO stage, DKO mice exhibited increased pro-inflammatory markers in adipose tissue, including CD95, and serum; *2)* total adipose tissue macrophages (ATMs) increased; *3)* CD95^+^CD86^-^ subset of ATMs also increased; and *4)* human aortic endothelial cells (HAECs) were activated (as determined by upregulated ICAM1 expression) when incubated with conditioned media from CD95^+^-containing DKO ATMs and human peripheral blood mononuclear cells-derived macrophages in comparison to respective controls. These results suggest that extended HFD in MHO mice promotes vascular inflammation and atherosclerosis *via* increasing CD95^+^ pro-inflammatory ATMs. In conclusion, we have identified a novel molecular mechanism underlying MHO transition to MUO with HFD. We have also found a previously unappreciated role of CD95^+^ macrophages as a potentially novel subset that may be utilized to assess pro-inflammatory characteristics of macrophages, specifically in adipose tissue in the absence of pro-inflammatory miR-155. These findings have provided novel insights on MHO transition to MUO and new therapeutic targets for the future treatment of MUO, MetS, other obese diseases, and type II diabetes.

## Introduction

Since the mid-1970s, the United States has seen a consistent rise in obesity in men and women, with rates of approximately 35% and 40%, respectively (1). Likewise, direct and indirect medical costs of obesity have steadily risen from a 39.3 billion dollar estimate in 1986 to 147 billion dollars in recent years (2, 3). Behind much of the high cost of obesity are the commonly resulting ailments, such as type 2 diabetes (T2DM), coronary heart disease, hypertension, dyslipidemia, atherosclerosis, non-alcoholic fatty liver disease (NAFLD), sleep apnea (4), osteoarthritis, gallbladder disease, stroke, several forms of cancer, and depression (2). However, not all obese individuals develop these associated metabolic dysfunctions. While the classical model of obesity (metabolically unhealthy obesity) is often associated with the comorbidities mentioned above, there exists a subset of obese individuals who do not present with these metabolic dysfunction-related comorbidities and are thus referred to as metabolically healthy obese (5).

Defining metabolically healthy obesity (MHO) has proven challenging due to a lack of consensus in the field (6, 7). As a result, determining the prevalence of MHO proves difficult. In 2017, a meta-analysis of 40 population-based MHO studies found the global rate to be an estimated 35% of obese individuals (8). We previously introduced an MHO mouse model, which maintained insulin sensitivity despite obesity. Additionally, our model did not meet the criteria for metabolic syndrome (MetS), which is the presence of at least three of five risk factors: enlarged waistline, hypertension, low high-density lipoprotein (HDL) levels, high triglyceride levels, and high fasting blood glucose level. Apolipoprotein E-knockout (ApoE^-/-^) mice are a well-accepted atherogenic mouse model (9). As we previously reviewed (10), the discovery of non-coding RNAs, including microRNAs (miRs), has revolutionized the way that we examine the genome, RNA products, and the regulation of transcription and translation. By facilitating mRNA degradation and translation repression, miRs regulate inflammatory responses, endothelial cell activation, atherosclerosis, obesity, non-alcoholic fatty liver disease (NAFLD), and other diseases (11). Our ApoE^-/-^/miR155^-/-^ (DKO) mice on high-fat diet (HFD) maintained normal plasma triglyceride levels, normal fasting blood glucose level, and had increased plasma HDL level compared with ApoE^-/-^ mice on normal chow diet (NC) and high-fat diet (HFD) as well as with DKO mice on NC (11). Several studies agree that, compared with metabolically unhealthy obesity (MUO), MHO is associated with a reduced risk for developing cardiovascular diseases (CVDs), type 2 diabetes mellitus (T2DM) (12, 13) and with lower mortality (14, 15). However, MHO has an increased risk compared with a metabolically healthy (MH) lean state, which remains the case no matter the stringency of criteria applied (16). Moreover, several studies demonstrate that MHO patients often switch to being MUO. This ranged from approximately 30 to 50% of MHO subjects and took place within approximately three to ten years (8, 17, 18). However, the underlying mechanisms for how MHO transition may occur are unknown.

It is well-documented that obesity is characterized by chronic, low-grade, sterile inflammation, and that macrophages are the predominant immune cells in adipose tissue (6, 19–21). In fact, human adipose tissue macrophages (ATMs) increase from between 5% and 10% in lean adipose tissue to between 40% and 50% in obese adipose tissue. Moreover, macrophages play a key role in adipose tissue inflammation *via* pro-inflammatory cytokine/adipokine secretion (22) and pro-inflammatory miR155-containing exosomes (23), which contribute to systemic inflammation (24). However, more studies are needed to decipher the many macrophage subtypes and their roles in systemic inflammation.

Fas, or CD95, is a member of the death receptor family, which also includes the tumor necrosis factor α (TNFα) cognate receptor, TNF receptor type 1 (TNFR1). As the family name suggests, Fas was initially discovered as a death receptor, specifically inducing apoptosis (25). However, its role in non-apoptotic inflammation has also been demonstrated (26–29). In fact, several papers document pro-inflammatory cytokine production (interleukin-1β (IL-1β), IL-6, IL-8, TNFα) *via* Fas signaling in adipocytes and monocytes/monocyte-derived macrophages (30–33). Moreover, Fas mRNA levels are significantly higher in circulating blood monocytes of obese subjects compared with lean subjects. Obese subject stratification into those with normal glucose tolerance versus those with T2DM showed that the latter expressed higher levels of Fas in monocytes (30). In addition, Fas protein showed a trend of increase in the adipose tissue of obese subjects compared with lean subjects, and was more highly expressed in adipose tissue of T2DM obese subjects compared with non-T2DM obese subjects (33). Furthermore, plasma TNFα levels were significantly reduced in myeloid-specific Fas-depleted mice on high-fat diet (HFD). Monocyte-derived Fas mRNA levels and circulating TNFα protein levels were positively correlated in human serum (33), supporting a link between Fas in myeloid cells and increased TNFα levels. These data support the idea of a pro-inflammatory role for Fas in obesity, with and without metabolic diseases. However, issue of whether CD95 serves as a marker for pro-inflammatory macrophages remains.

In our current study, we hypothesized that MHO is a transition phase toward MUO, and that inflammation driven by ATMs is a novel mechanism for this transition. We found that, with extended HFD (24 weeks of HFD), MHO mice became MUO, as judged by increased atherosclerosis. At the MHO stage, DKO mice exhibited increased pro-inflammatory markers in adipose tissue, including CD95, and serum. We found that total adipose tissue macrophages were increased, and that a new CD95^+^CD86^-^ subset of adipose tissue macrophages (ATMs) was increased. These results highlight a role for CD95^+^ macrophages as a player in MHO and its transition to MUO. We hope that these findings will propel the field of MHO forward toward greater understanding as well as lead to and enhance clinical therapy.

## Methods

### Animal Care

All animal experiments were performed in accordance with the Institutional Animal Care and Use Committee (IACUC) guidelines and were approved by the IACUC of Lewis Katz School of Medicine (LKSOM) at Temple University. Apolipoprotein E (ApoE, B6.129P2-*ApoE^tm1Unc^*/J, stock no. 002052) knockout mice, microRNA-155 (miR-155, B6.Cg-*Mir155^tm1Rsky^*/J, stock no. 007745) knockout mice, and wild-type (WT) mice were of a C57BL/6J background, and were purchased from the Jackson Laboratory (Bar Harbor, ME, United States). Mice were housed under controlled conditions in the LKSOM Animal Facility, where they had *ad libitum* access to standard chow diet/HFD, water, and were subject to a 12-h light-dark cycle. DKO mice were generated as previously reported (11) by crossing ApoE^-/-^ mice with miR155^-/-^ mice. Mice were age-matched and gender-specific in all experiment groups, unless otherwise stated. At eight weeks old, mice either remained on normal chow diet (10.7% fat, 23.9% protein, 5.1% fiber, 58.7% carbohydrate/other, 200ppm cholesterol; Labdiet 5001) or switched to HFD [20% (w/w) fat, 17.4% protein, 5% fiber, 49.9% carbohydrate/other, 2027 ppm cholesterol (0.15% (w/w) cholesterol); TestDiet AIN-76A] (34) for 12 weeks or 24 weeks, specified in each experiment.

### Mouse Genotyping

Mouse genotype was confirmed using end-point polymerase chain reaction (PCR) on genomic DNA obtained from mouse tail sample. Briefly, DNA was extracted using 50μl of extraction solution on tail samples, followed by incubation at 95^o^C for 30 min. Afterwards, 50μl of stabilization solution was added (Extracta™ DNA Prep for PCR, QuantaBio, cat. No. 97065-350, VWR, Radnor, PA). PCR was then performed (**Supplementary Table 1**), followed by 1.5% agarose gel electrophoresis. The ethidium bromide-containing gel was then imaged by ultraviolet using Foto® analyst image system (Fotodyne, Fisher Scientific, Hartland, Wisconsin).

### Gonadal White Adipose Tissue Single-Cell Suspension

Gonadal white adipose tissue (gWAT) was isolated and mechanically digested, followed by enzymatic digestion with collagenase type II (Sigma, cat. No. C6885, St. Louis, MO) at 37^o^C. Following filtration steps and centrifugation, the remaining immune cell-containing stromal vascular fraction (SVF) was stained in preparation for flow cytometry.

### Flow Cytometry

Following single-cell suspension, SVF cells were stained with live/dead dye (ThermoFisher, Waltham, MA) for 30 min at room temperature in the dark. After washing with Hank’s balanced salt solution (HBSS) (Corning, Corning, NY) supplemented with 2% fetal bovine serum (FBS) (GE Life Sciences, Marlborough, MA), Fc receptor block (ThermoFisher) was added to cells. Following a 5-min incubation, cells were incubated with surface antibodies for 15 min at room temperature in the dark. Intracellular markers: After washing, cells were fixed (ThermoFisher) for 30 min, washed and permeabilized (ThermoFisher) for 15 min. Cells were then incubated with antibodies for 20 min at room temperature in the dark, followed by washing (**Supplementary Table 2**). Data was collected using BD LSRII flow cytometer and DIVA software (BD Biosci., Woburn, MA). Data were analyzed using FlowJo (BD Biosci.).

### Cytokine Array

Mouse blood was allowed to clot for 4 h at room temperature. After centrifuging for 15 min at 2,000xg, supernatant (i.e., serum) was carefully obtained and stored at −80^o^C until further use. Cytokine array experiment was conducted according to the manufacturer’s protocol (R&D, Minneapolis, MN, cat. No. ARY028). Blots were imaged *via* chemiluminescence method; X-ray film exposure in a dark room and development with SRX-101A medical film processor (Konica, Tokyo, Japan). Protein levels were quantified using ImageJ software.

### Murine Aortic Single-Cell Suspension

After perfusion, whole aortas were isolated and collected in DMEM-low medium (GE Life Sciences) supplemented with 20% FBS. Aortas were rinsed in PBS (Corning), dissected, and then enzymatically digested with cocktail consisting of FBS, HEPES (Gibco, Gaithersburg, MD), hyaluronidase type I-S (Sigma), collagenase types I (Sigma) and XI (Sigma) at 37^o^C for 30 min. Next, the suspension was filtered, then washed and resuspended in HBSS supplemented with 2% FBS, before staining for flow cytometry.

### Human Aortic Endothelial Cell Culture

Human aortic endothelial cells (HAECs) (Lonza, Basel, Switzerland) were cultured on gelatin-coated flask in Medium 199 (GE Life Sciences) supplemented with FBS, PSA (ThermoFisher), ECGS (endothelial cell growth serum, Corning), and heparin (Sigma). The medium was changed every two days and cells were passaged at 70% confluency, not exceeding more than two subcultures.

### Adipose Tissue Macrophage Culture

SVF cells were cultured in murine macrophage differentiation medium [RPMI 1640 medium supplemented with 25 ng/ml macrophage colony-stimulating factor (R&D), HEPES (Gibco), sodium pyruvate (Sigma), non-essential amino acids (Gibco), GlutaMAX (Gibco)] for six to seven days at 5x10^5^ cells per well in six-well plates (Falcon, VWR).

### Human Adipose Tissue

We acknowledge Dr. Zsolt Bagi for generously donating IRB-approved de-identified human pericardial adipose tissue (**Supplementary Table 3**).

### Human Peripheral Blood Mononuclear Cell Isolation and Culture

Whole blood from healthy male donors (**Supplementary Table 4**) was collected in anticoagulant solution [aqueous solution of sodium citrate (Fisher Scientific), citric acid (Sigma), and dextrose (Sigma)]. Blood was then gently layered onto Histopaque-1077 (Sigma), followed by centrifugation at room temperature. The peripheral blood mononuclear cell (PBMC)-containing phase was gently isolated then washed with PBS, followed by ammonium-chloride-potassium (ACK) lysis. Following centrifugation, PBMCs were resuspended in human macrophage differentiation medium [RPMI 1640 medium supplemented with 50 ng/ml human macrophage colony-stimulating factor (R&D), HEPES, sodium pyruvate, non-essential amino acids, glutamax] for six to seven days at 1x10^6^ cells per well in six-well plates.

### Human Peripheral Blood Mononuclear Cell Stimulation Assay

After six to seven days of culture, human PBMCs remained unstimulated or were stimulated with TNFα (10 ng/ml) (R&D) for 24 h, followed by assessment of Fas expression.

### Human Aortic Endothelial Cells Activation With CD95^+^ Macrophage-Conditioned Medium

HAECs were plated at 5x10^5^ cells per well in six-well plates for 24 h. Following, HAECs were treated with macrophage-conditioned medium, with macrophage differentiation medium, or with endothelial cell medium for 24 h. HAECs were then assessed for EC activation *via* flow cytometry.

### RNA Extraction and Quantification

Briefly, 100 to 200 mg of liquid nitrogen-frozen adipose tissue was homogenized using mortar and pestle, followed by the addition of QIAzol lysis reagent (Qiagen, Hilden, Germany). After homogenate was brought to room temperature, chloroform (Sigma) was added, then solution was vigorously shaken. Following centrifugation and aqueous phase retrieval, 100% ethanol (PHARMACO-AAPER, Thermal Scientific) was added and mixed. Next, following the manufacturer’s protocol (miRNeasy Mini Kit, Qiagen), ethanol-aqueous phase solution was added to RNeasy Mini columns and subjected to a series of buffer washes and centrifugation steps. RNA was resuspended in nuclease-free water. RNA quality and concentration were determined using NanoDrop 2000 (ThermoFisher).

### RNA Reverse Transcription and Quantitative Real-Time PCR

Per the manufacturer’s instruction, total RNA was reverse transcribed to generate complementary DNA (cDNA) using the miScript II RT Kit (Qiagen). Briefly, template RNA in tubes containing buffer, Nucleics Mix, Reverse Transcriptase Mix and RNase-free water was reverse transcribed at 37^o^C for 60 min and 95^o^C for five min to generate cDNA.

Quantitative PCR (qPCR) was performed using the StepOnePlus PCR System (Applied Biosystems, Foster City, CA), following preparation with miScript SYBR Green PCR Kit for Use with miScript Primer Assays (Qiagen). Primers for human and mouse miRNA-155 and the housekeeping gene RNU6 (miScript Primer Assay) were purchased from Qiagen. Cycling conditions were as follows: 40 cycles at 95^o^C for 15 min, 94^o^C for 15 s, 55^o^C for 30 s, and 70^o^C for 30 s. Data were analyzed using the delta-delta Ct method.

### Atherosclerotic Lesion Analysis

Following perfusion with PBS, mouse aortas were excised and fixed overnight in 4% paraformaldehyde (Sigma). Next, aortas were placed in 20% sucrose (Sigma) for 24 h. Aortas were then stored in PBS at 4°C. For *en face* staining, aortas were stained in Sudan IV (Sigma) for 40 min at 37°C, followed by incubation in 70% isopropanol for five min. Afterwards, aortas were opened longitudinally and imaged using AxioCam camera mounted to Stemi 2000-C microscope (Carl Zeiss Inc., Jena, Germany).

### Protein Extraction and Western Blot

Adipose tissue and aortas were sonicated in an aqueous sample buffer consisting of sodium dodecyl sulfate (SDS, Sigma), Tris-hydrochloride [Tris (Fisher Scientific)-HCl (Sigma)], glycerol (ThermoFisher), PBS, EDTA, phenylmethylsulfonyl fluoride (PMSF, Sigma), and cOmplete Protease Inhibitor Cocktail (Sigma). Following centrifugation and retrieval of the protein-containing supernatant, protein concentration was determined using the Pierce BCA Protein Assay Kit (ThermoFisher). For gel electrophoresis, 15 to 30 μg of protein were loaded into wells and run for 90 min. Next, proteins were transferred onto polyvinylidene fluoride (PVDF) membrane (Bio-Rad, Hercules, CA) for 90 min at 400mA. Following Ponceau S (Sigma) staining, the membrane was blocked with 5% non-fat milk (Lab Scientific) for 1 h at room temperature and washed. The membrane was then incubated with primary antibody in non-fat milk or bovine serum albumin (BSA, Gemini Bioproducts, West Sacramento, CA) overnight at 4°C (**Supplementary Table 5**). Afterwards, the membrane was washed and incubated with horseradish peroxidase (HRP)-linked secondary antibody at room temperature between 30 and 120 min (**Supplementary Table 5**). Membranes were washed and incubated in enhanced chemiluminescence substrate (ThermoFisher) for five min prior to imaging. Next, protein bands were imaged on X-ray film (AGFA, Mortsel, Belgium) after development with SRX-101A medical film processor (Konica).

### Statistical Analysis

Statistical analyses were performed using the GraphPad Prism software. Two-tailed Student’s *t*-test was used for statistical comparison between two groups. One-way ANOVA with Tukey Multiple Comparison test was used for three or more groups. Data presented as mean ± SEM (standard error of the mean). Statistical significance was defined as *p*<0.05.

## Results

### MicroRNA-155 Transcripts Are Decreased in White Adipose Tissue of Obese Wild-Type Mice Compared With White Adipose Tissue of ApoE^−/−^ Mice

**Figure 1:**
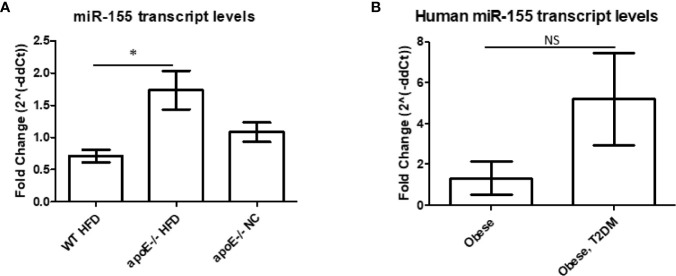
MicroRNA-155 transcripts are decreased in white adipose tissue (WAT) of obese wild-type (WT) mice compared with WAT of apolipoprotein E (ApoE)^-/-^ mice. **(A)** Mice were kept on either normal chow (NC) or switched to high-fat diet (HFD) at 8 weeks old and fed for an additional 12 weeks. ApoE^-/-^ NC (n=6); apoE^-/-^ HFD (n=7); WT HFD (n=4). *p < 0.05. **(B)** Obese patients with or without type 2 diabetes mellitus (T2DM). Donor information detailed in Table 3. Obese (n=2), Obese, T2DM (n=4). *p* > 0.05. NS, not significant.

Our previous data showed that miR-155 was significantly increased in the aortas of the well-established atherosclerotic mouse model, ApoE^-/-^ mice, following 12 weeks of HFD versus normal chow (11). In addition, following 12 weeks of HFD, DKO (ApoE^-/-^/miR155^-/-^) mice (i.e., our MHO model) showed significant reduction of atherosclerotic plaque deposition in aortas compared with ApoE^-/-^ mice. DKO mice also presented with obesity without insulin resistance (11, 35), as shown by a normal glucose tolerance test (GTT) and insulin tolerance test (ITT) (11). These results led to the classification of these mice as MHO. In our current study, to corroborate our finding of miR-155 suppressing obesity, we found reduced miR-155 transcript levels in the classical obese model (WT mice on 12 weeks of HFD) compared with atherosclerotic mice (**Figure 1A**), which correlated well with our data mining findings in **Figure 3D** of our previous report (11). Furthermore, we found that human pericardial adipose tissue from obese patients with T2DM showed a trend of increased miR-155 transcripts compared with obese patients without T2DM (**Figure 1B**) (see additional information in the Discussion). Taken together, these results demonstrate that WT mice on HFD and obese patients without T2DM have reduced miR-155 levels compared with atherogenic mice and obese patients with T2DM. Since pro-inflammatory miR-155 suppresses adipogenic transcription factors such as peroxisome proliferator-activated receptor gamma (PPARγ), and CCAAT/enhancer-binding protein alpha (C/EBPα), miR155 reduction and/or deficiency allow MHO establishment as we reported (11, 36).

### ApoE^−/−^/miR155^−/−^ (DKO) Mice Exhibit Resurgent Atherosclerosis Following Extended HFD Feeding

**Figure 2:**
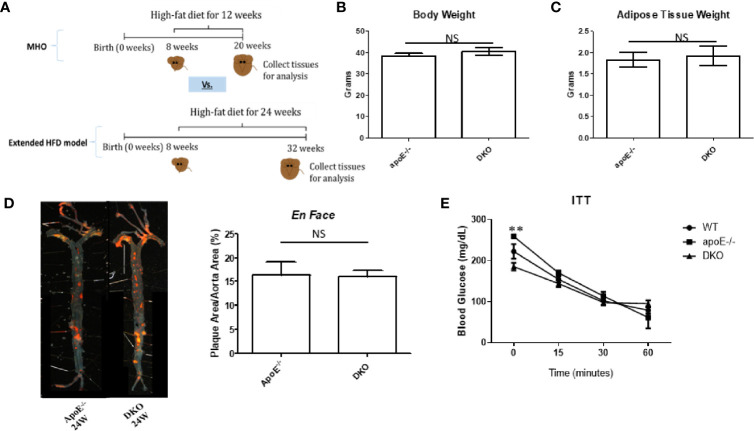
miR155-/-/ApoE-/- double gene knock-out (DKO) mice exhibit resurgent atherosclerosis following extended high fat diet (HFD) feeding. **(A)** Extended HFD experiment design. DKO mice at MHO stage are fed on HFD for 12 weeks beginning at 8 weeks. DKO mice at extended HFD stage (when MUO develops) are fed on HFD for 24 weeks beginning at 8 weeks. **(B)** Total body weight (grams); ApoE-/- (n=15), DKO (n=12). **(C)** Gonadal WAT (grams); apoE^-/-^ (n=10), DKO (n=11). **(D)** *En Face* analysis of whole aortas; male ApoE^-/-^ (n=5); male DKO (n=8). Previous data showed that aortas from 12 weeks of HFD-fed DKO mice compared with ApoE^-/-^ mice had significantly less plaque deposition (PMID: 27856635). Here, following 24 weeks of HFD, both ApoE^-/-^ and DKO mice have significant plaque deposition. (E) Insulin Tolerance Test (ITT); WT (n=3), ApoE^-/-^ (n=2), DKO (n=3). **p<0.01. NS, not significant.

As earlier mentioned, at least 30% of obese subjects eventually develop MUO (17, 18). MUO is associated with an inflammatory environment perpetuated by ATMs (36, 37). We sought to uncover, first, whether our MHO model transitioned to MUO over time; and, second, whether DKO mice at the MHO stage have increased pro-inflammatory macrophages and cytokine secretion. In order to define whether MHO mice can transition to MUO, we extended HFD from 12 to 24 weeks (**Figure 2A**) and determined whether MHO mice developed MUO as judged by atherosclerosis development. We found that there were no significant differences in total body weight or in gWAT weight between ApoE^-/-^ and DKO mice following 24 weeks of HFD (**Figures 2B, C**). This is in contrast to the significant differences in total body weight as well as gWAT weight following 12 weeks of HFD between the two genotypes (11). Previously, we reported that following 12 weeks of HFD, DKO mice showed a significant reduction in aortic plaque deposition compared with ApoE^-/-^ mice (11). Here, that difference was lost as shown by increased atherosclerosis in DKO mice following 24 weeks of HFD. DKO mice exhibited extensive aortic plaque deposition, signifying a resurgent atherosclerosis phenotype (**Figure 2D**). Moreover, there were no significant differences in blood glucose levels in response to insulin challenge between ApoE^-/-^ and DKO male mice, which were similar to our previous findings following 12 weeks of HFD. However, DKO mice exhibited a lower blood glucose level at baseline, before insulin challenge (**Figure 2E**). These data show that MHO status was lost over time as DKO mice developed MUO following 24 weeks of HFD and that miR-155 deficiency in ApoE^-/-^ background with extended HFD promotes obesity.

### High-Fat Diet-Fed DKO Mice (Metabolically Healthy Obesity Stage) Exhibit White Adipose Tissue Inflammation

**Figure 3:**
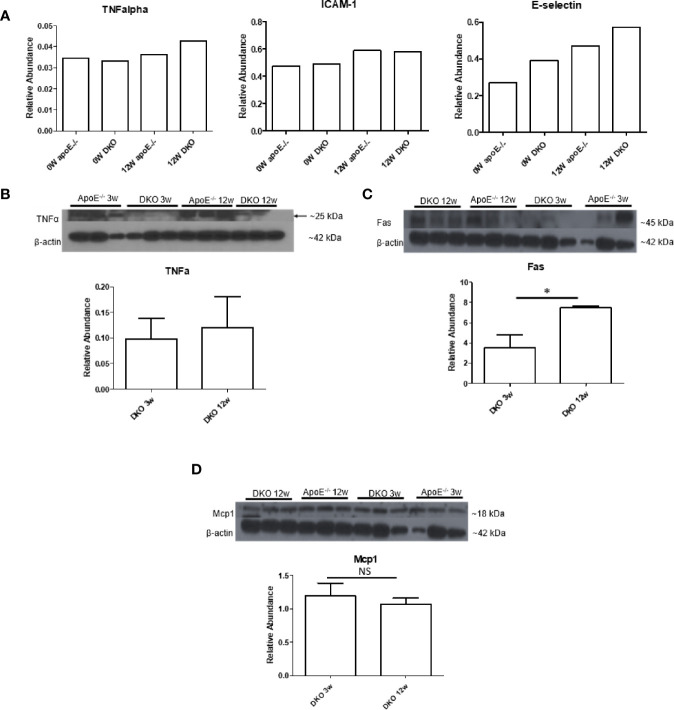
Pro-inflammatory tumor necrosis factor-a (TNFa) and Fas are increased while leptin and Mcp1 show no change. **(A)** 0W: 8-week old male mice on normal chow (NC). 12W: 20-week old male mice that began HFD at 8 weeks old and continued for 12 weeks; n=3 per sample. Each group is composed of samples from 3 mice combined into 1 sample for analysis. **(B–D)** 3W: 11-week old male mice that began HFD at 8 weeks old and continued for 3 weeks. 12W: 20-week old male mice that began HFD at 8 weeks old and continued for 12 weeks; n=3 per group. *p<0.05. NS, not significant.

After establishing that MHO mouse on extended HFD becomes MUO and is therefore an appropriate model to study MHO switch to MUO, we assessed whether DKO mice at the MHO stage exhibited a pro-inflammatory profile, which would contribute to these mice developing MUO over time. Cytokine array analysis showed that the pro-inflammatory cytokine, TNFα, and the endothelial cell (EC) activation markers, soluble intercellular adhesion molecule 1 (Icam1) and soluble E-selectin, were induced in the serum of DKO mice, showing a trend of increase with HFD feeding (**Figure 3A**). Previously, we reported that the pro-inflammatory adipokines, leptin and resistin, were increased in the plasma of DKO mice compared with ApoE^-/-^ mice after 12 weeks of HFD (11). We also previously found that resistin was significantly increased in DKO gonadal WAT (gWAT) compared with WT gWAT and ApoE^-/-^ gWAT (36). However, the changes in leptin were not significant in the gWAT of WT, ApoE^-/-^ and DKO mice after 12 weeks of HFD (**Supplementary Figure 1**).

We next examined the pro-inflammatory marker, TNFα, and Fas, which were previously reported to be elevated in obese adipose tissue (33). We also examined monocyte chemoattractant protein 1 (Mcp1/chemokine C-C motif ligand 2, CCL2), a chemokine that recruits macrophages to tissues. We found that TNFα showed higher expression at 12 weeks of HFD compared with 3 weeks of HFD, though this difference was not statistically significant. Fas, but not Mcp1, was significantly increased in gWAT of DKO mice following 12 weeks of HFD versus three weeks of HFD (**Figures 3B–D**). These data indicate that although DKO mice at the MHO stage are protected from or have delayed atherosclerosis development, a miR155-independent pro-inflammatory and pro-atherogenic environment is present.

### High-Fat Diet-Fed DKO Mice (Metabolically Healthy Obesity Stage) Exhibit Increased Macrophages in White Adipose Tissue

**Figure 4:**
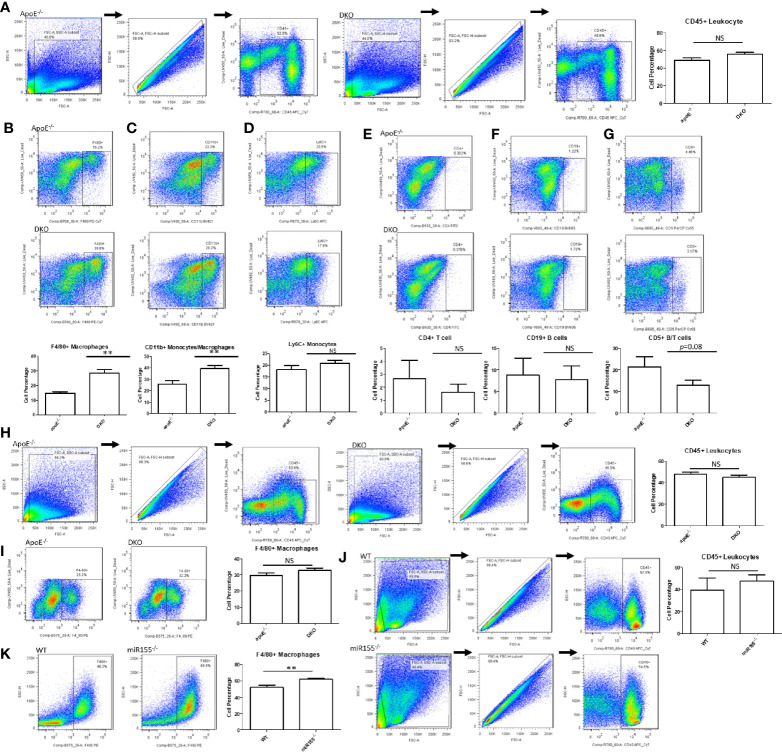
Macrophages and monocytes are increased in miR155-deficient, high fat diet (HFD)-fed male mice in the presence or absence of ApoE background. Mice were fed on HFD from 8 weeks old to 20 weeks old. **(A)** Male mice: Total leukocytes were defined as CD45+. Macrophages/monocytes were defined as **(B)** CD45+F4/80+ or **(C)** CD45+CD11b+. **(D)** Inflammatory monocytes were defined as CD45+Ly6C+. **(E)** CD4+ T lymphocytes were defined as CD45+CD4+. **(F)** B lymphocytes were defined as CD45+CD19+. **(G)** B/T lymphocyte subsets were defined as CD45+CD5+; ApoE^-/-^ (n=7), DKO (n=11). **(H)** Female mice: Total leukocytes were defined as CD45+. **(I)** Macrophages were defined as CD45+F4/80+; ApoE^-/-^ (n=5), DKO (n=4). **(J)** Male mice: Total leukocytes were defined as CD45+. **(K)** Macrophages were defined as CD45+F4/80+; WT (n=8), miR155^-/-^ (n=9). **p<0.01. NS, not significant.

Next, we assessed the percentages of various immune cell types within the gWAT. In male ApoE^-/-^ and DKO mice, there was no significant difference in total leukocyte percentage (**Figure 4A**). Likewise, we found no significant differences in Ly6C^+^ monocytes (38), CD4^+^ T cells (39), or CD19^+^ B cell populations (40) (**Figures 4D–F**). In contrast, F4/80^+^ macrophages and F4/80^+^CD11b^+^ monocyte/macrophages were significantly increased in DKO gWAT compared with ApoE^-/-^ gWAT (**Figures 4B, C**). Interestingly, we found that CD5^+^ T lymphocytes/B lymphocytes showed a trend of decrease in DKO mice (**Figure 4G**), a finding that correlates well with miR-155’s role in promoting lymphoproliferative B cell disorder (41, 42). When we assessed the total leukocytes and F4/80^+^ macrophages in female mice, we saw that there were no significant differences (**Figures 4H, I**). Moreover, we did not find a significant change in total leukocyte population between miR155^-/-^ gWAT and WT gWAT (**Figure 4J**). However, when we examined F4/80^+^ macrophages in male miR155^-/-^ gWAT compared with WT gWAT, we saw a significant increase in the former (**Figure 4K**), suggesting that miR-155 deletion supports macrophage proliferation and/or infiltration.

As previously mentioned, an increase in macrophage number is a hallmark of obesity (20). In addition, macrophages exist as varying subsets in adipose tissue, most notably pro-inflammatory M1 macrophages and anti-inflammatory M2 macrophages (43). We next assessed whether these macrophages could be broadly classified as pro-inflammatory or anti-inflammatory. We found that there were no significant changes in pro-inflammatory (M1) ATMs or anti-inflammatory (M2) ATMs in ApoE^-/-^ and DKO male and female mice as well as WT and miR155^-/-^ male mice (**Supplementary Figure 2**). Interestingly, we found that the majority of the ATMs expressed both M1 and M2 markers in male ApoE^-/-^ and DKO mice. Previous papers have reported similar findings, where double-positive macrophages are hypothesized to be pro-inflammatory (44, 45). These results—suggesting M1 and M2 double-positive macrophages as a feature of ATMs in obesity conditions—are well correlated with our recent report (37).

### High-Fat Diet-Fed DKO Mice (Metabolically Healthy Obesity Stage) Exhibit Increased CD95^+^CD86^−^ Macrophage Subset in White Adipose Tissue

**Figure 5:**
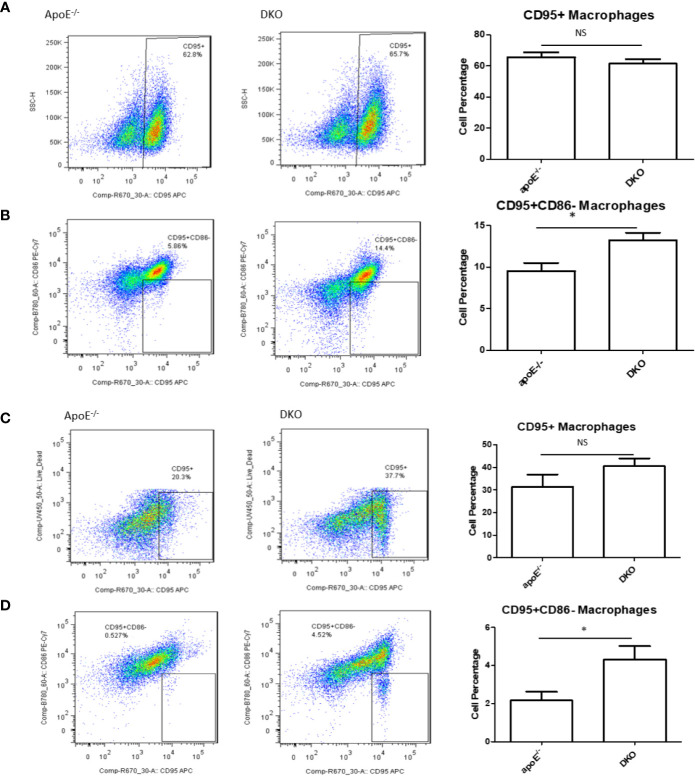
A novel subset of CD95^+^CD86^-^ macrophages is increased in the white adipose tissues of DKO versus that of ApoE^-/-^ mice on high-fat diet. Mice were fed on HFD from 8 weeks old to 20 weeks old. Macrophages were defined as CD45^+^F4/80^+^ and then evaluated for the markers, CD95 and CD86. **(A, B)** Male mice: ApoE^-/-^ (n=5), DKO (n=4). **(C, D)** Female mice: ApoE^-/-^ (n=5), DKO (n=4). *p<0.05. NS, not significant.

In our previous publication, we found that pro-inflammatory macrophage markers in metabolic disease were lacking compared with markers for CVD and infectious diseases (46), thereby showing the need for a novel marker. Earlier, we showed that Fas was increased in DKO mice over time with HFD feeding. We examined whether CD95^+^ (Fas) macrophages in gWAT may be involved as a player in MHO mice. We found that while there was no significant difference in total CD95^+^ macrophages between ApoE^-/-^ and DKO male and female mice at MHO stage (**Figures 5A, C**), there was a significant increase in the CD95^+^CD86^-^ subset of CD95^+^ macrophages in both male and female DKO mice compared with ApoE^-/-^ mice (**Figures 5B, D**). However, there was no difference in the CD95^+^CD86^-^ macrophage population of ApoE^-/-^ and DKO male mice fed on NC for 20 weeks (**Supplementary Figure 3**). These data identify a potential novel subset of CD95^+^ macrophages that could play a role in MHO development, maintenance or progression.

We also assessed CD95^+^ macrophages in WT and miR155^-/-^ mice. We found that CD95^+^ macrophages were lower in miR155^-/-^ gWAT compared with WT gWAT, showing that the WT gWAT may have a more pro-inflammatory environment than miR155^-/-^ gWAT (**Supplementary Figure 4A**). In contrast, CD95^+^CD86^-^ macrophage population in WT versus miR155^-/-^ male mice did not show a robust population or significant difference (**Supplementary Figure 4B**). However, in extended HFD (24 weeks of HFD), we see that miR155^-/-^ gWAT had significantly more CD95^+^ macrophages than WT mice, unlike the 12-week HFD time point (**Supplementary Figure 4C**), which emphasized the roles of extended HFD. Additionally, while miR155^-/-^ mice exhibited fewer CD95^+^CD86^-^ macrophages than WT mice (**Supplementary Figure 4D**), these mice had a higher percentage of CD95^+^CD86^-^ macrophages at this extended time point compared with 12-week HFD. We recently reported that many of 28 T cell co-stimulatory receptors (CSRs), such as CD40, 4–1BBL, TL1A, CD30L, SLAM, CD48, SEMA4A, B7–1 (CD80), B7–2 (CD86), and CD155, are significantly upregulated in M1 macrophage polarization (47). This suggests a possibility that missing single CSR CD86, macrophages could still be polarized into pro-inflammatory macrophages such as CD95^+^CD86^-^ macrophages as demonstrated here; and that although Fas/CD95 induces apoptosis, CD40 expressed in M1 macrophages may rescue pro-inflammatory macrophages from CD95-mediated apoptosis, as reported in B cells (48).

The analyses of the expressions of 1376 innate immune genes in the CD95 KO microarray (NIH-NCBI-Geo Datasets database GSE111244) showed that 34 out of 1376 (2.5%) innate immune genes from the Innate Immune Database (https://www.innatedb.com/) were significantly downregulated (fold changes |log2|>1, *p*<0.05) compared with WT control cells (**Supplementary Figure 5A**). Further analysis of the downregulated innate immune genes in CD95 KO with the Ingenuity Pathway Analysis (IPA) showed that the top eight pathways of inflammation, including Th1 pathway, NF-ĸB signaling, systemic lupus erythematosus in B cells, cardiac hypertrophy signaling, production of nitric oxide and reactive oxygen species, neuroinflammation, systemic lupus erythematosus in T cells, and hepatic fibrosis signaling, were significantly downregulated (z score ≤1) compared with WT controls (**Supplementary Figure 5B**). Taken together, these results have demonstrated that CD95 may promote inflammation signaling in CD95^+^ macrophages, which were well correlated with our findings in ATM in gWAT in DKO mice and others’ reports (33, 49).

### Metabolically Healthy Obesity Aortas Exhibit Higher Expression of IL-1β and Fas Than miR155^−/−^ Aortas, and Metabolically Healthy Obesity Aortas Have No Reduction in Pro-Inflammatory Mediators Compared With Those of ApoE^−/−^ Mice

**Figure 6:**
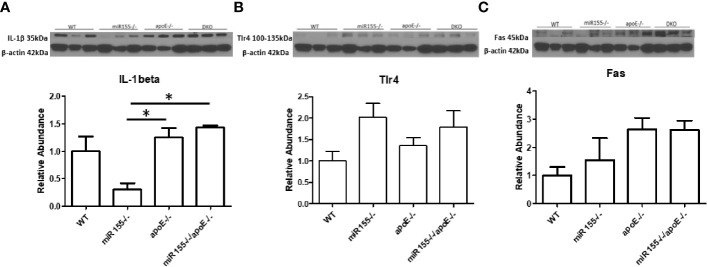
There are no differences in the expressions of three pro-inflammatory markers such as IL-1b, TLR4 and Fas in DKO aortas compared with ApoE-/- aortas following HFD. The expressions of IL-1b and Fas are significantly increased in DKO aortas compared with miR155-/- single KO aortas. A-C. Male mice were fed on HFD for 12 weeks from 8 weeks old to 20 weeks old. **(A)** Il-beta. **(B)** Tlr4. **(C)** Fas; n=3 per group. *p<0.05.

A pro-inflammatory function for Fas has been discovered in both atherosclerosis and obesity (33, 49). We next examined whether Fas and other pro-inflammatory molecules were changed in the aorta at the MHO stage compared with ApoE^-/-^ aortas. We found that the expression of IL-1b was increased in DKO aortas compared with miR155^-/-^ single KO aortas. DKO aortas showed no reduction in pro-inflammatory mediators, IL-1β, TLR4 (50) [previous studies show a link with atherosclerosis in ApoE^-/-^ mice (51, 52)], and Fas compared with ApoE^-/-^ aortas (**Figure 6**). To determine whether aortic expressions of Fas and TLR4 were partially contributed by aortic monocytes and macrophages, the mouse aortic single cell RNA-Seq (scRNA-Seq) data were analyzed on the Single Cell^Beta^ Portal database (https://singlecell.broadinstitute.org/single_cell) of the Broad Institute of MIT and Harvard. The results in **Supplementary Figure 6** showed that aortic expressions of Fas and TLR4 were partially contributed by aortic monocytes and macrophages (53). Taken together, these findings show that MHO mouse aortas have higher expressions of IL-1b than miR155^-/-^ aortas; and MHO aortas are not less pro-inflammatory compared with ApoE^-/-^ mouse aortas. Of note, we previously reported that IL-17A promotes endothelial cell activation but not atherosclerosis (54); that deficiency of mRNA-decaying protein tristetraprolin (TTP) in bone marrow cells promote strong systemic inflammation but not atherosclerosis in low-density lipoprotein receptor (LDLR)-deficient mice (55). Thus, during MHO transition to MUO, inflammatory status and atherosclerosis may not always progress at the same pace. In addition, we recently proposed a new working model that miR-155-suppressed “secondary wave inflammatory state (SWIS)” may be characteristic of MHO transition to MUO (36).

### The Culture Medium of DKO Stromal Vascular Fraction Containing CD95+ ATMs Promotes Aortic Endothelial Cell Activation

**Figure 7:**
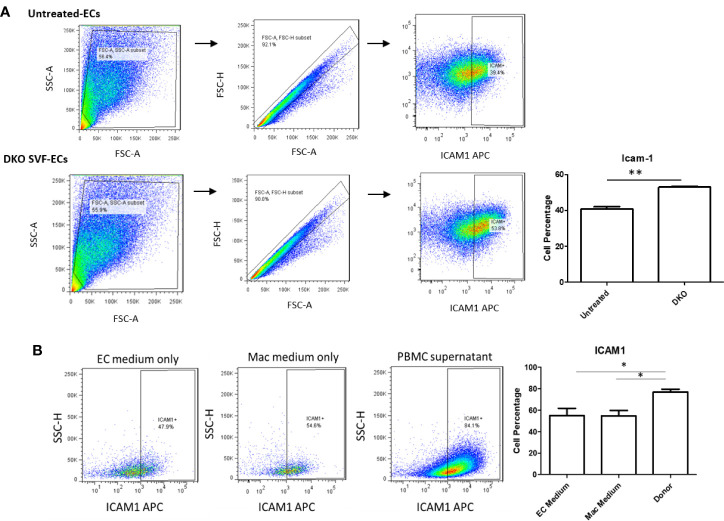
DKO SVF and human macrophage-conditioned medium promote HAEC activation. **(A)** Stromal vascular fraction (SVF) from 20-week old DKO male mice on NC was cultured overnight in macrophage differentiation medium. Centrifuged supernatant was incubated with human aortic ECs (HAECs) for 24 hours. Untreated HAECs were incubated with EC medium; untreated (n=3), DKO-treated (n=3). **(B)** Isolated human peripheral blood mononuclear cells (PBMCs) were grown for 7 days in macrophage differentiation medium. Afterwards, cultured HAECs were incubated with centrifuged supernatant (macrophage-conditioned medium) for 24 hours. HAECs in EC medium group were incubated with EC medium only for 24 hours and HAECs in Mac medium group were incubated with macrophage medium only for 24 hours; EC medium (n=11), Mac medium (n=11), Cond Mac medium (n=12). *p<0.05; **p<0.01.

**Figure 8:**
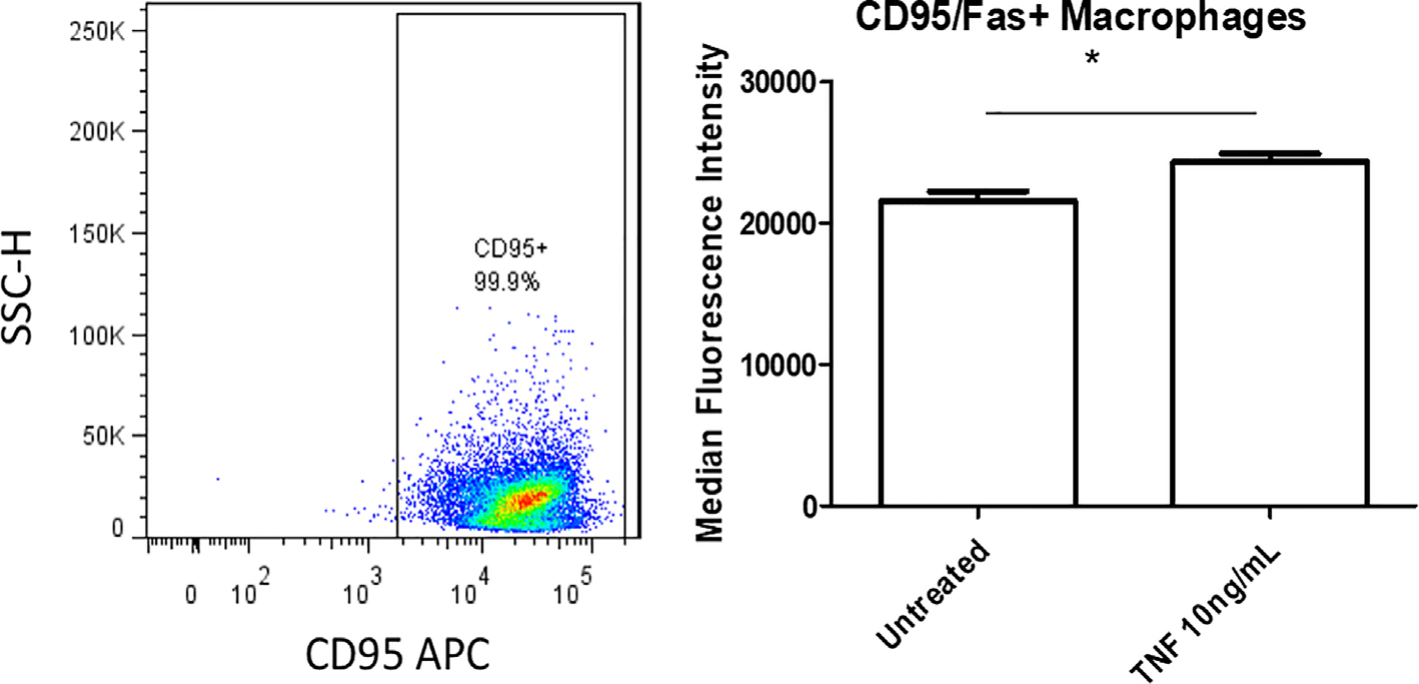
TNFα-treated human peripheral blood mononuclear cells (PBMCs) show increased Fas expression. Isolated human PBMCs were grown for 7 days in macrophage differentiation medium. Cells were treated with TNFα (10ng/mL) for 24 hours. Untreated was incubated with fresh medium; Untreated (n=4), TNFα (n=4). *p<0.05.

We next assessed whether CD95^+^ macrophages could promote EC activation, which is the early stage of atherosclerotic development (56, 57). Previous studies’ results support a role for ATMs in atherosclerosis development and metabolic vascular disease (23, 58). We first performed a data mining analysis on a dataset of single cell RNA-Seq of mouse SVF from mouse adipose tissue deposited in the scRNA-Seq database (the Single Cell^Beta^ Portal database) in the Broad Institute of MIT and Harvard to verify CD95 (Fas) expression on the monocytes and macrophages. As shown in **Supplementary Figure 7A**, FAS transcript expressions were detected in the scRNA-Seq dataset of the monocytes and macrophages in the SVF of adipose tissues, which were well correlated with our FACS data in **Figure 5**. In addition, proinflammatory cytokines TNF-α and IL-1β transcript expressions were also detected in the monocytes and macrophages in the mouse adipose tissue SVF (**Supplementary Figures 7B, C**), which were well correlated with the reported proinflammatory function of FAS in obesity (33, 49). Moreover, as shown in **Supplementary Figure 8**, the expressions of FAS, TNFα and IL-1β were also found in the scRNA-Seq datasets from the four monocyte clusters in human peripheral blood (59) in the scRNA-Seq database (the Single Cell^Beta^ Portal database). Then, using cultured human aortic endothelial cells (HAECs), we found that HAECs incubated with DKO SVF-conditioned medium (which contains CD95^+^ macrophages) for 24 h resulted in increased EC activation, shown by a significant increase in ICAM1 expression compared with untreated ECs (**Figure 7A**). Moreover, incubating HAECs with human macrophage-conditioned medium resulted in increased ICAM1 expression compared with HAECs incubated with control EC medium or macrophage differentiation medium only (**Figure 7B**). Our findings were well correlated with others’ reports (60). Seminal studies of obesity have helped us to understand that macrophages secrete TNFα, which further enhances lipolysis, thereby driving metabolic dysfunction (61). Moreover, when this occurs in the visceral adipose tissue (VAT), collateral damage to nearby organs, such as liver and pancreas, can lead to metabolic dysfunction of these organs (62, 63). Regarding the mechanism of MHO, a previous report showed that TNFα can increase Fas expression (64). Additionally, we showed that TNFα was induced in our DKO mouse WAT at MHO over time. We found that TNFα-treated human peripheral blood mononuclear cells (PBMCs) resulted in increased Fas expression detected by flow cytometry (**Figure 8**). Taken together, these results suggest that in addition to CD95^+^ monocytes/macrophages found in aortas and peripheral blood by scRNA-Seq data (**Supplementary Figures 7** and **8**), DKO ATMs containing CD95^+^ macrophages may secrete pro-inflammatory cytokines, including TNFα, and induce Fas expression *via* potential autocrine manner in ATMs and activate aortic endothelial cells. Future work is needed to determine whether CD95^+^ macrophages are essential for the culture medium of DKO SVF to promote HAEC activation, and to assess what cytokines from DKO SVF-containing CD95^+^ macrophages are responsible for activating HAECs.

## Discussion

**Figure 9:**
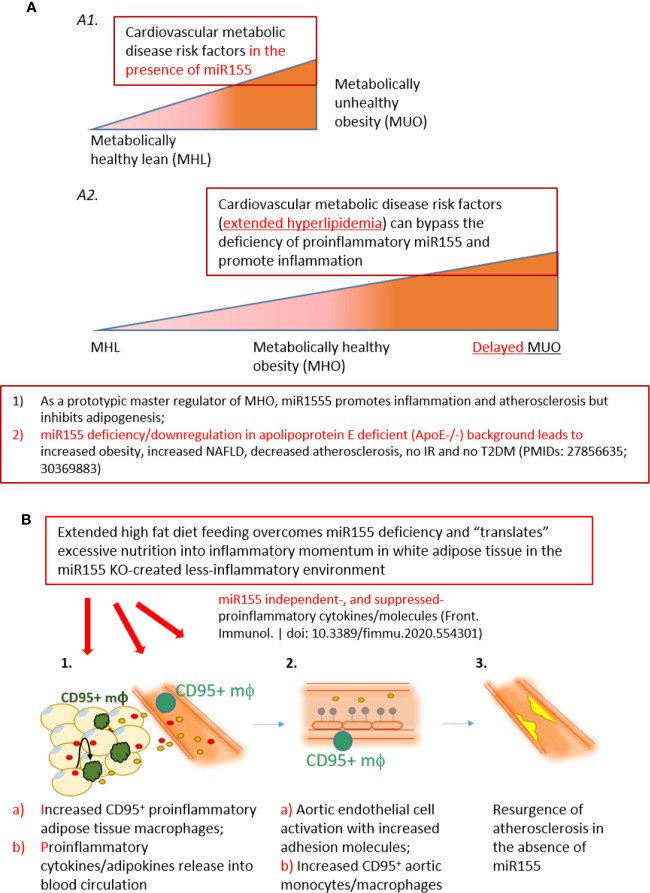
A new working model: Extended high fat diet feeding overcomes the deficiency of proinflammatory miR155, generates CD95+ adipose tissue macrophages and secretes miR155 independent-, or miR155-suppressed proinflammatory cytokines/adipokines (second wave of inflammation), promotes aortic endothelial cell activation and increases atherosclerosis, which makes MHO transition to delayed MUO. **(A)** MHO is an intermediate stage in the development of delayed MUO. **(*A1*)** in the presence of wild-type miR155, cardiovascular metabolic disease risk factors accelerate the progression of MHO from metabolically healthy lean; **(*A2*)** In the absence of miR155, development of MUO need to go through an intermediate stage of MHO. **(B)** **(*B1*)** Adipocyte (pale yellow with light blue nuclei)-secreted and macrophage-secreted TNFα (red dots) may promote CD95+CD86- macrophage phenotype (green). This macrophage subset together with peripheral blood CD95+ monocytes/macrophages may secrete pro-inflammatory cytokines and chemokines into the circulation (yellow dots), which in turn induce aortic EC activation, shown by ICAM1 upregulation (***B2***, gray dots). The CD95+ monocytes/macrophages in aortas may also secrete proinflammatory cytokines and contribute to EC activation **(*B2b*)**. **(C)** Over time, EC activation progresses towards atherosclerosis development (***B3***, bright yellow). Red dots: TNFα. Yellow dots: pro-inflammatory cytokines/chemokines/other molecules.

Despite its recognition since the 1980s (65), the mechanisms underlying why some obese individuals maintain MHO status as well as how MHO individuals progress toward MUO remain understudied. Several correlations have been determined to help explain what prompts MHO switch to MUO. For example, Schröder *et al*. reported that increasing waist-to-hip ratio, waist circumference and BMI are factors (66), in addition to increasing age (6). Moreover, MHO patients more likely to transition to MUO were women (67). Although a molecular mechanism for MHO switch to MUO status (68, 69) has not been discovered, genetic mechanisms have been proposed, since MUO has been linked to genes (70). Likewise, a number of mechanisms have been suggested to explain how some instances of obesity can be MH. Some include reduced immune cell infiltration in adipose tissue; conserved insulin sensitivity; MHO patients’ proclivity to deposit lipids in subcutaneous adipose tissue (SAT) depot along with lower visceral adipose tissue (VAT) and lower ectopic fat (in skeletal muscle and liver). Additionally, greater level of fitness and exercise have been proposed (15). Furthermore, lower levels of C-reactive protein, TNFα, and IL-6 were reported in patients with MHO compared with MUO individuals (71–76). Additionally, MHO individuals had higher adiponectin (anti-inflammatory adipokine) level (71, 77), lower white blood cell count, and lower plasminogen activator inhibitor-1 (PAI-1) level (71). We previously reported the first mouse model of MHO with miR155 deficiency (11) (**Figure 9A**). As a prototypic master regulator, miR155 promotes inflammation and atherosclerosis but inhibits the adipogenesis of WATs. Thus, the deficiency of miR155 in ApoE^-/-^ background leads to increased obesity, increased non-alcoholic fatty liver disease (NAFLD), decreased atherosclerosis, no insulin resistance and no type II diabetes mellitus (T2DM) (11). Given what is already known, it is clear that there exists the need to improve our understanding of MHO and the underlying mechanisms of its transition to MUO.

To this end, we have utilized our miR155^-/-^/ApoE^-/-^ (DKO) male mice fed on HFD as a suitable MHO model (11). Not only did we find a role for miR-155 in MHO in mice, we found that miR155 transcripts showed a trend of increase in pericardial adipose tissue from obese-type 2 diabetic patients compared with obese patients. A limitation of our study is the difference in visceral adipose tissue depot: *in vivo* mouse studies utilized gonadal adipose tissue, while our human adipose tissue was pericardial. Although literature comparing the pericardial and gonadal/epididymal fat depots, specifically, were not found, both fat depots exhibit inflammatory and/or metabolic dysfunction parameters (78–80). Future work is needed to verify a similar inflammatory profile between the two adipose tissue depots. Several studies report that approximately one-third to one-half of MHO individuals develop MUO, showing that MHO may not be a stable condition for a significant portion of obese patients. We therefore aimed to better understand how MHO transitions to MUO (36). We determined whether MHO mice develop MUO with extended HFD, which mimics the MHO patient continuing an HFD lifestyle. We saw that over time, extended HFD-fed MHO model developed atherosclerosis, although aortic plaque deposition was not significantly different between ApoE^-/-^ and DKO mice at 24 weeks (**Figure 2**). This demonstrated MHO transition to MUO, which has been delayed due to miR155 deficiency (**Figure 9A2**). We recently proposed a new concept of second wave of inflammation status, in which a proinflammatory master regulator such as miR155 promotes the first wave of inflammation but inhibits the second wave inflammation so that when it is deficient, the second wave of inflammation is increased (36). In our recent report (Front. Immunol. doi: 10.3389/fimmu.2020.554301), we further found that proinflammatory cytokine blockages induce inflammatory regulators. In the circulation, we found that TNFα and soluble forms of E-selectin and Icam1 were induced in DKO mice at the MHO stage (**Figure 3A**). It is possible that TNFα levels may be significantly attributed to gWAT, which we showed produced increasing amounts of TNFα over time (**Figure 3B**). Additionally, the soluble forms of E-selectin and ICAM1 are signatures of EC activation, suggesting that ECs at the MHO stage may be activated (81, 82) (**Figure 9B**). We next assessed the pro-inflammatory environment (if any) in DKO mice at the MHO stage and found that TNFα and Fas expression in gWAT were increased in DKO mice at the MHO stage compared with DKO mice at an earlier stage of HFD feeding. The levels of Mcp1, however, were unchanged, suggesting that macrophage recruitment was not changed, at least at this stage (83) (**Figures 3B–D**). We found that macrophages were significantly increased in male DKO versus ApoE^-/-^ mice as well as miR155^-/-^ vs WT mice, as assessed by flow cytometry (**Figures 4B, C, K**). Moreover, analysis of the macrophages as pro-inflammatory (CD45^+^F4/80^+^CD86^+^) or anti-inflammatory (CD45^+^F4/80^+^CD206^+^) showed no difference between ApoE^-/-^ and DKO male and female mice. No difference was observed between WT and miR155^-/-^ mice as well (**Supplementary Figure 2**). Our recent publication highlights that metabolic disease markers for pro-inflammatory macrophages are lacking, especially when compared with markers for CVD and infectious diseases (Table 3a in our recent publication) (46). Therefore, novel markers are needed. Fas (or CD95) is well-known for its pro-apoptotic role (25) but also has a non-apoptotic function as a pro-inflammatory mediator in cells such as macrophages (30–33). We examined whether CD95^+^ macrophages are increased in MHO mice. We found that while total CD95^+^ macrophages were not increased in MHO mice compared with ApoE^-/-^ mice, the subset CD95+CD86- was (**Figures 5A, B**). This was also observed in female DKO mice compared with ApoE^-/-^ mice, albeit with a smaller percentage compared with male mice (**Figure 5D**). These differences were unsurprising since female DKO mice exhibited a significant yet smaller difference in body and gWAT weights compared with female ApoE^-/-^ mice in our previous report (11). In WT and miR155^-/-^, we observed that CD95^+^ macrophages were significantly reduced in miR155^-/-^ mice following 12 weeks of HFD and that there was no difference in CD95^+^CD86^-^ macrophage population (**Supplementary Figures 4A, B**). With extended HFD feeding (24 weeks HFD), we saw that CD95^+^ macrophages significantly increased in miR155^-/-^ mice compared to WT mice (**Supplementary Figure 4C**). While CD95^+^CD86^-^ macrophages are significantly lower in miR155^-/-^ mice compared with the population in WT mice after extended HFD, the overall numbers of CD95^+^CD86^-^ miR155^-/-^ macrophages between 12 weeks and 24 weeks have increased; from approximately 2% to 12.5%, respectively (**Supplementary Figures 4B, D**). Additional experiment is needed to determine the extent to which CD95+ macrophage deletion can block MHO-MUO transition. Previous study showed that deletion of CD95 in adipocytes did not affect mouse weight, but led to improved insulin sensitivity, reduced plasma free fatty acid level, and reduced plasma pro-inflammatory cytokine IL-6. Also, adipocyte-specific CD95 deletion in mice had decreased Mcp1 and Cd11b mRNA levels, showing macrophages might be reduced (33).

Moving beyond, we found that there were no differences in the expression of pro-inflammatory IL-1β, TLR4 and Fas between the well-established atherosclerotic model, ApoE^-/-^ mice, and our MHO mice (**Figure 6**). In other words, MHO mice maintained a pro-inflammatory environment, despite very minimal plaque manifestation. We and collaborators recently report a similar finding that deficiency of mRNA-decaying protein, TTP, in bone marrow cells promotes strong systemic inflammation but not atherosclerosis in LDLR^-/-^ mice (55). Macrophages may exert their pro-inflammatory effects in a paracrine manner or *via* cell-cell contact. Using the culture medium of DKO SVF, which includes CD95^+^ macrophages, as well as using macrophage differentiation culture medium of PBMCs from human blood (84), we found increased ICAM1 expression on HAECs compared with respective controls (**Figures 7A, B** **)**. Additionally, we found that treatment with TNFα, which was increased in DKO WAT, for 24 h increased Fas (CD95) detection in PBMCs, showing that TNFα in WAT of DKO model may increase the expression of CD95^+^ on macrophages (**Figure 8**). Our single cell RNA-Seq data analysis results showed that CD95, TNF-α and IL-1β are co-expressed in CD95^+^ monocytes and macrophages in aortas and peripheral blood (**Supplementary Figures 7** and **8**), and CD95 expressions are increased in MHO aortas in hyperlipidemia conditions (**Figure 6**). We performed Cytoscape database analysis (https://cytoscape.org/) on four pro-inflammatory molecules identified in our MHO model here (FAS, TNFα, IL-1β, TLR4), as well as on two adipogenesis regulators (C/EBP, PPARγ), and on two adipokines (leptin, resistin) in our reports (11, 36), which indicates that these pro-inflammatory pathways in MHO interplay with lipid storage and adipogenesis (**Supplementary Figure 9**). Taken together, our study suggests that CD95^+^CD86^-^ ATMs may represent a novel subset for driving MHO transition to MUO *via* secretion of proinflammatory adipokines and cytokines in the absence of miR155, as shown in our new working model (**Figure 9**). Practically, it may represent the degree of transition to MUO in a clinical setting. Furthermore, this subset defines a previously unappreciated role for CD95^+^ macrophages in obesity, which may have similar pro-inflammatory roles in other disease conditions.

## Data Availability Statement

The datasets presented in this study can be found in online repositories. The names of the repository/repositories and accession number(s) can be found in the article/**Supplementary Material**.

## Ethics Statements

The studies involving human participants were reviewed and approved by the Temple University Institutional Review Board (IRB) and the Institutional Biosafety Committee (IBC). Human samples were de-identified patient samples. The animal study was reviewed and approved by the Temple University Laboratory Animal Resources (ULAR) and the Institutional Animal Care & Use Committee (IACUC).

## Author Contributions

CJ carried out the experiments, data analysis, and drafted the manuscript. CDIV, HS, YSh, YSu, and YL aided in the data collection. FS, KX, GN, PF, ZB, XJ, EC, and HW provided material input. XY supervised the experimental design and data analysis, and edited the manuscript. All authors contributed to the article and approved the submitted version.

## Conflict of Interest

The authors declare that the research was conducted in the absence of any commercial or financial relationships that could be construed as a potential conflict of interest.

## Supplementary Material

Supplementary Figure 1 Leptin is not significantly changed in DKO mice. 20-week old male mice were fed on HFD for 12 weeks beginning at 8 weeks old; n=3 per group. *p*>0.05. NS, not significant.

Supplementary Figure 2 There is no significant difference in CD86+ (M1) or CD206+ (M2) ATMs. Pro-inflammatory macrophages were defined as CD45+F4/80+CD86+. Anti-inflammatory macrophages were defined as CD45+F4/80+CD206+. **(A)** male ApoE^-/-^ (n=4), DKO (n=6). **(B)** male WT (n=10), miR155^-/-^ (n=8). **(C)**. female ApoE^-/-^ (n=5), DKO (n=4). *p*>0.05. NS, not significant.

Supplementary Figure 3 A novel subset of macrophages shows no significant difference in DKO vs ApoE^-/-^ male mice on NC. Male mice were fed on NC and analyzed at 20 weeks old. ApoE^-/-^ (n=5), DKO (n=7). *p*>0.05. NS, not significant.

Supplementary Figure 4 CD95+ macrophage percentage increases with HFD feeding. **(A, B)** Male mice were fed on HFD from 8 weeks old to 20 weeks old. **(A)** Macrophages were defined as CD45+F4/80+CD95+. **(B)** Macrophages were defined as CD45+F4/80+CD95+CD86-; WT (n=5), miR155^-/-^ (n=6). **(C, D)** Male mice were fed on HFD from 8 to 32 weeks old. **(C).** Macrophages were defined as CD45+F4/80+CD95+. **(B)** Macrophages were defined as CD45+F4/80+CD95+CD86-; WT (n=7), miR155^-/-^ (n=10). *, *p*<0.05. NS, not significant.

Supplementary Figure 5 CD95 (Fas) promotes inflammation signaling pathways. **(A)** The expressions of 34 genes out of 1376 innate immune genes from the Innate Immune Database (https://www.innatedb.com/) with the methods reported in our recent paper (https://www.frontiersin.org/articles/10.3389/fimmu.2020.554301/abstract) were decreased in the FAS knock-out microarray dataset (NIH-NCBI-Geo Datasets database GSE111244, FAS KO versus control cells). **(B)** The Ingenuity Pathway Analysis (IPA) with the Fas KO-decreased genes showed that eight inflammation signaling pathways were significantly downregulated (Z score ≥ 1 or Z score ≤ -1).

Supplementary Figure 6 Fas mRNA transcripts are found in mouse aortic monocyte 1, monocyte 2 and macrophages. The data mining analyses were performed on the Single Cell RNA-Seq database of the Broad Institute of MIT and Harvard (Single Cell^Beta^ Portal; https://singlecell.broadinstitute.org/single_cell/study/SCP289/single-cell-analysis-of-the-normal-mouse-aorta-reveals-functionally-distinct-endothelial-cell-populations#study-summary, PMID: 31146585). **(A)** Fas expressions in monocytes and macrophages were circled in red in the Scatter; **(B)** Fas expressions in monocytes and macrophages were boxed in red in the Distribution; **(C)** Toll-like receptor 4 (TLR4) expressions in monocytes and macrophages were also boxed in read in the Distribution.

Supplementary Figure 7 The expressions of Fas, TNFα, and IL-1b are found in all the four clusters of monocytes/macrophages in mouse adipose tissue stromal vascular fraction (SVF). The data mining analyses were performed on the Single Cell RNA-Seq database of the Broad Institute of MIT and Harvard (Single Cell^Beta^ Portal; https://singlecell.broadinstitute.org/single_cell/study/SCP708/mouse-adipose-stromal-vascular-fraction#study-summary). **(A)** Fas expressions in monocytes and macrophages were boxed in red in the Scatter; **(B)** TNFα expressions in monocytes and macrophages were boxed in red in the Distribution; **(C)** IL-1b expressions in monocytes and macrophages were also boxed in read in the Distribution.

Supplementary Figure 8 The expressions of Fas, TNFα, and IL-1b are found in all the four clusters of monocytes/macrophages in human blood. The data mining analyses were performed on the Single Cell RNA-Seq database of the Broad Institute of MIT and Harvard (Single Cell^Beta^ Portal; https://singlecell.broadinstitute.org/single_cell/study/SCP43/atlas-of-human-blood-dendritic-cells-and-monocytes#study-summary; PMID: 28428369). **(A)** Fas expressions in monocytes and macrophages were boxed in red in the Scatter; **(B)** TNFα expressions in monocytes and macrophages were boxed in red in the Distribution; **(C)** IL-1b expressions in monocytes and macrophages were also boxed in read in the Distribution.

Supplementary Figure 9 Extended high fat diet (HF) feeding-upregulated proinflammatory regulators including Fas (CD95), TNFα, IL-1b, and TLR4 overcome miR155 deficiency and promote resurgence of atherosclerosis. We reported that the expressions of C/EBP, PPARg, leptin (Lep), and resistin are upregulated in HF-fed MHO mice (PMIDs: 27856635; 30369883); and we also found that the expressions of CD95, TNFα, IL-1b and TLR4 are increased in HF-fed MHO mice in this study. The Cytoscape analyses (https://cytoscape.org/; the network data integration, analysis and visualization database) indicate that pro-inflammatory and pro-immune, lipid storage and white adipose tissue differentiation pathways are interplayed and promote resurgence of atherosclerosis and MHO transition to MUO.
