# Supplementary figures and images for: A Novel Subset of CD95+ Pro-Inflammatory Macrophages Overcome miR155 Deficiency and May Serve as a Switch From Metabolically Healthy Obesity to Metabolically Unhealthy Obesity
Source: Front Immunol. 2021 Jan 7;11:619951. doi: 10.3389/fimmu.2020.619951 (PMC7817616; doi:10.3389/fimmu.2020.619951)

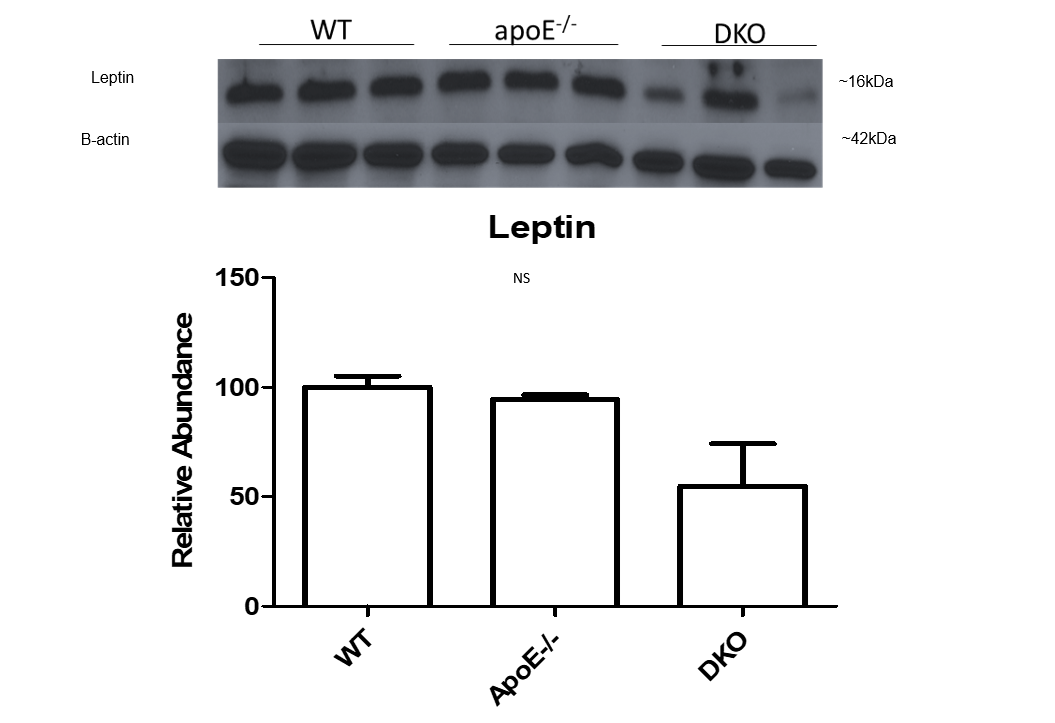

Supplement: Supplementary Figure 1 — Leptin is not significantly changed in DKO mice. 20-week old male mice were fed on HFD for 12 weeks beginning at 8 weeks old; n=3 per group. p>0.05. NS, not significant. [file Image_1.tif]

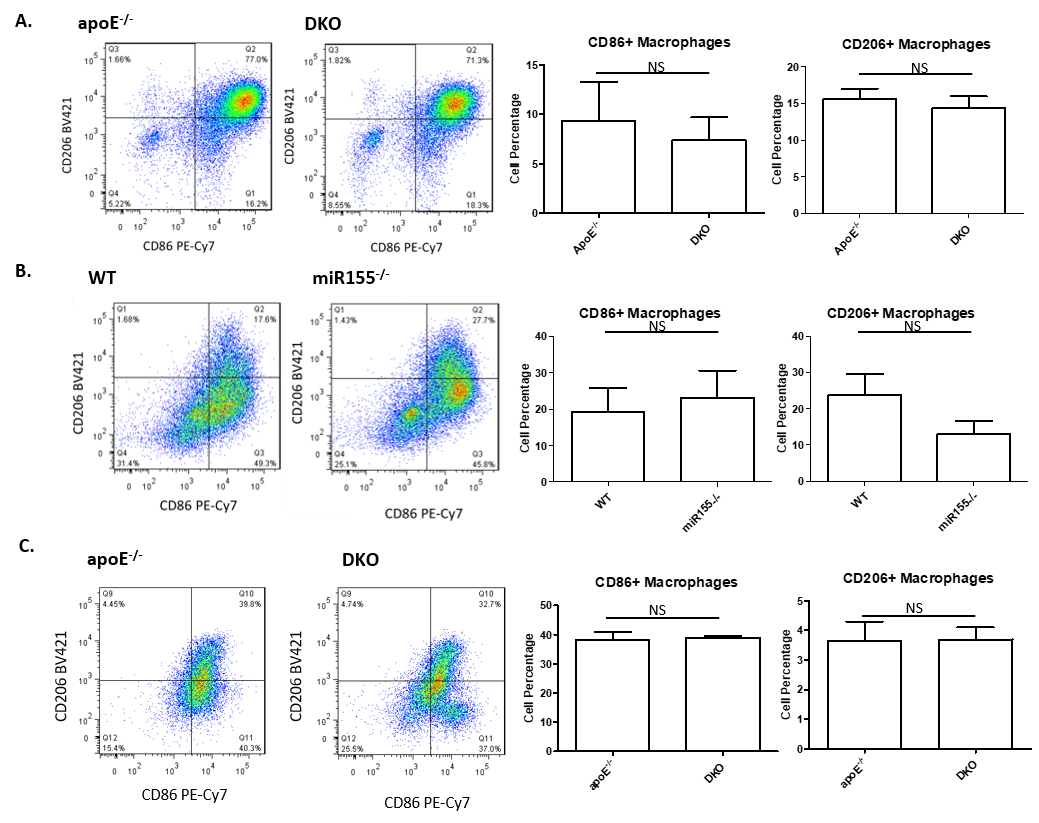

Supplement: Supplementary Figure 2 — There is no significant difference in CD86+ (M1) or CD206+ (M2) ATMs. Pro-inflammatory macrophages were defined as CD45+F4/80+CD86+. Anti-inflammatory macrophages were defined as CD45+F4/80+CD206+. (A) male ApoE-/- (n=4), DKO (n=6). (B) male WT (n=10), miR155-/- (n=8). (C). female ApoE-/- (n=5), DKO (n=4). p>0.05. NS, not significant. [file Image_2.tif]

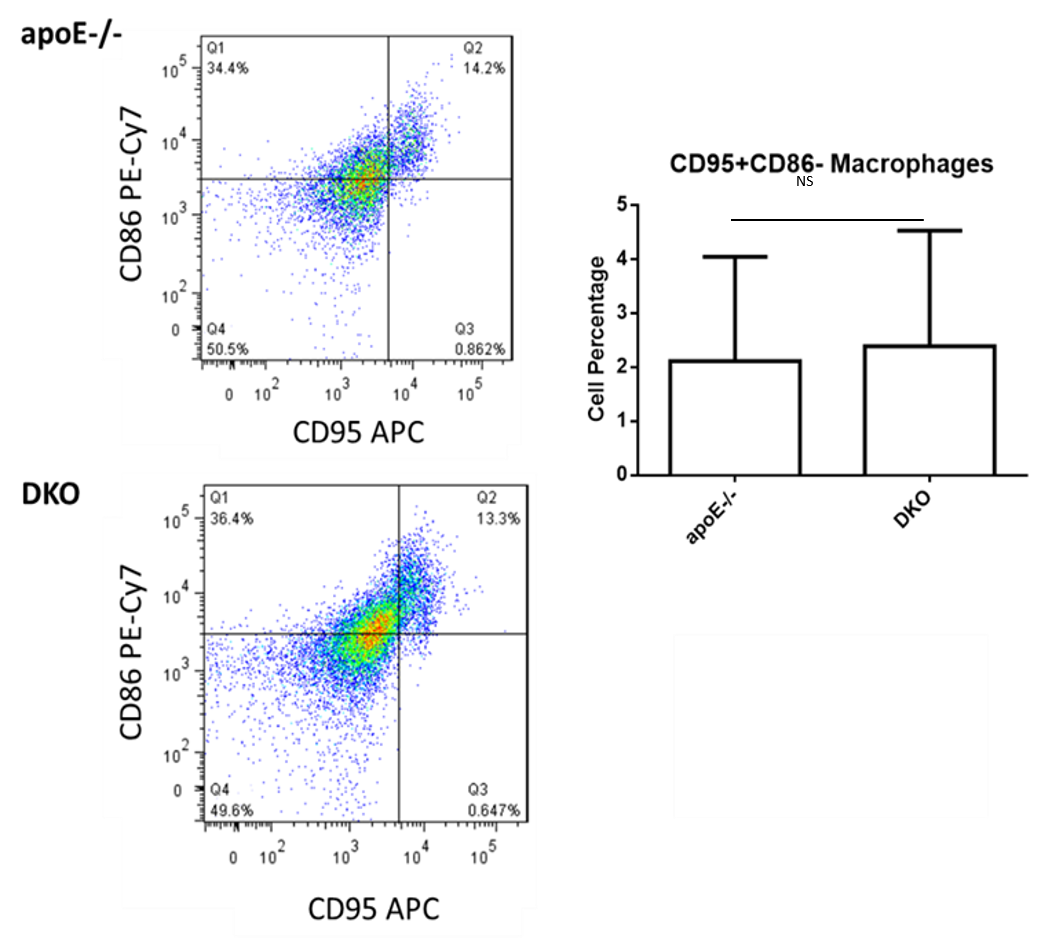

Supplement: Supplementary Figure 3 — A novel subset of macrophages shows no significant difference in DKO vs ApoE-/- male mice on NC. Male mice were fed on NC and analyzed at 20 weeks old. ApoE-/- (n=5), DKO (n=7). p>0.05. NS, not significant. [file Image_3.tif]

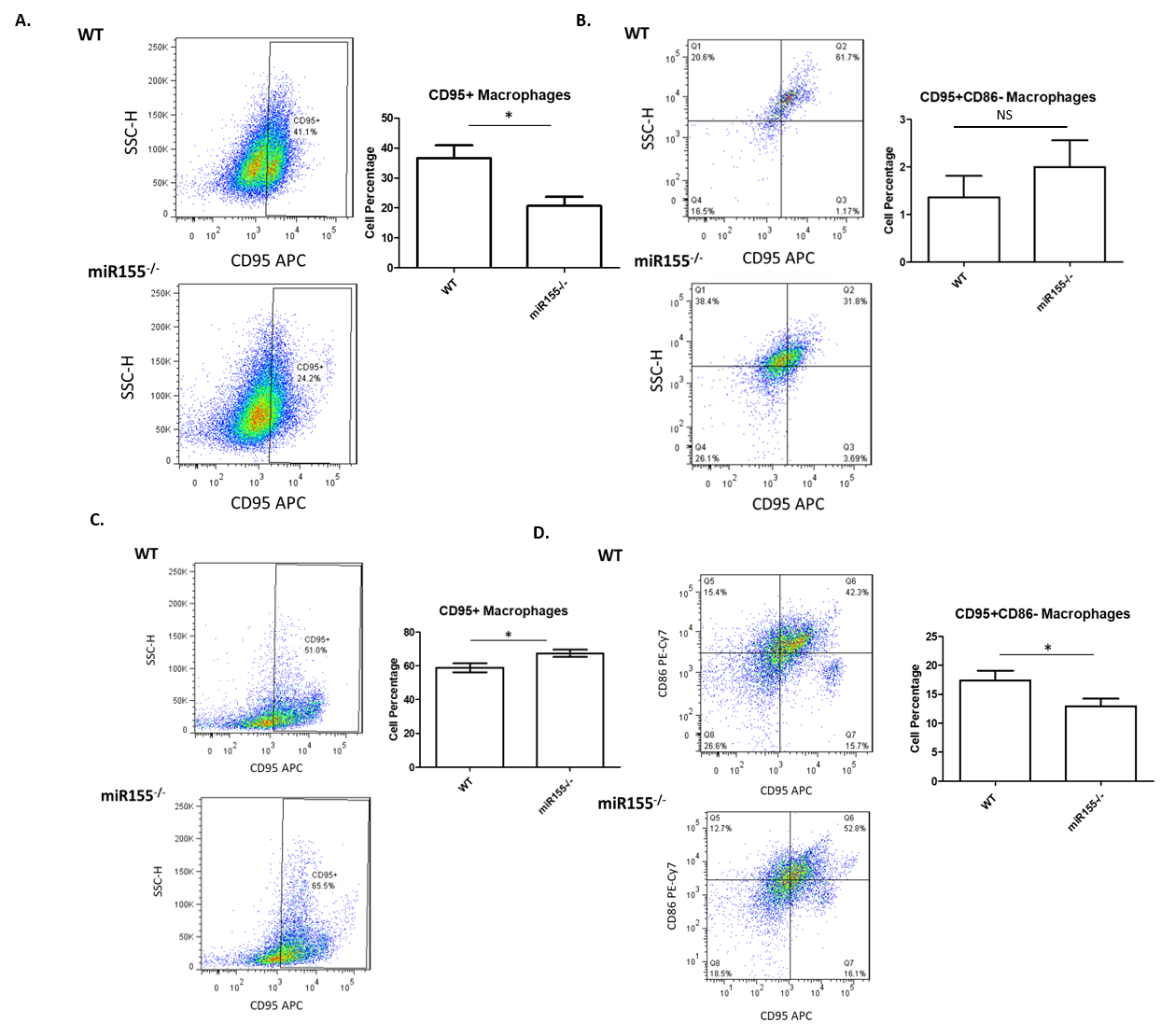

Supplement: Supplementary Figure 4 — CD95+ macrophage percentage increases with HFD feeding. (A, B) Male mice were fed on HFD from 8 weeks old to 20 weeks old. (A) Macrophages were defined as CD45+F4/80+CD95+. (B) Macrophages were defined as CD45+F4/80+CD95+CD86-; WT (n=5), miR155-/- (n=6). (C, D) Male mice were fed on HFD from 8 to 32 weeks old. (C). Macrophages were defined as CD45+F4/80+CD95+. (B) Macrophages were defined as CD45+F4/80+CD95+CD86-; WT (n=7), miR155-/- (n=10). *, p<0.05. NS, not significant. [file Image_4.tif]

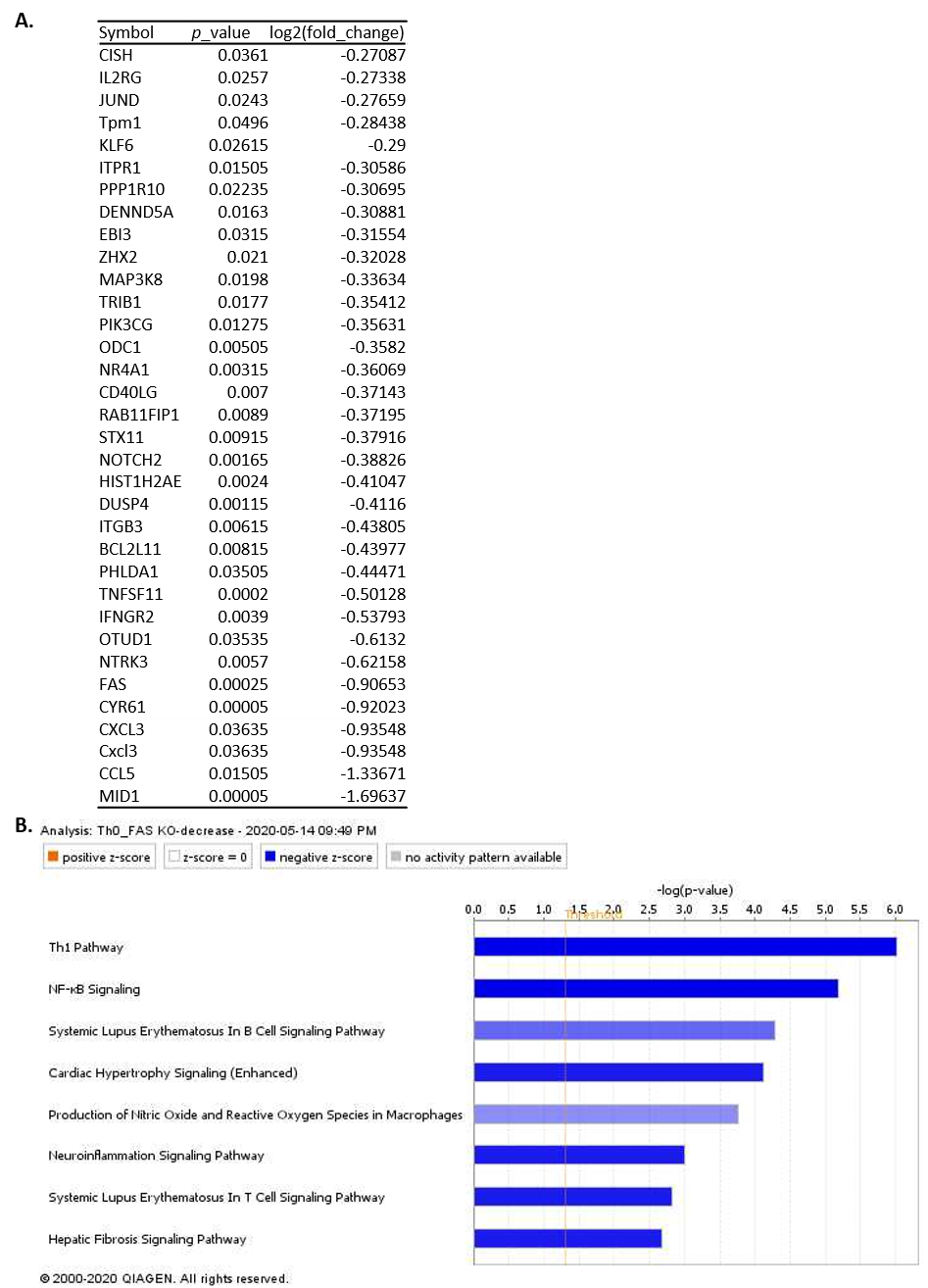

Supplement: Supplementary Figure 5 — CD95 (Fas) promotes inflammation signaling pathways. (A) The expressions of 34 genes out of 1376 innate immune genes from the Innate Immune Database (https://www.innatedb.com/) with the methods reported in our recent paper (https://www.frontiersin.org/articles/10.3389/fimmu.2020.554301/abstract) were decreased in the FAS knock-out microarray dataset (NIH-NCBI-Geo Datasets database GSE111244, FAS KO versus control cells). (B) The Ingenuity Pathway Analysis (IPA) with the Fas KO-decreased genes showed that eight inflammation signaling pathways were significantly downregulated (Z score ≥ 1 or Z score ≤ -1). [file Image_5.tif]

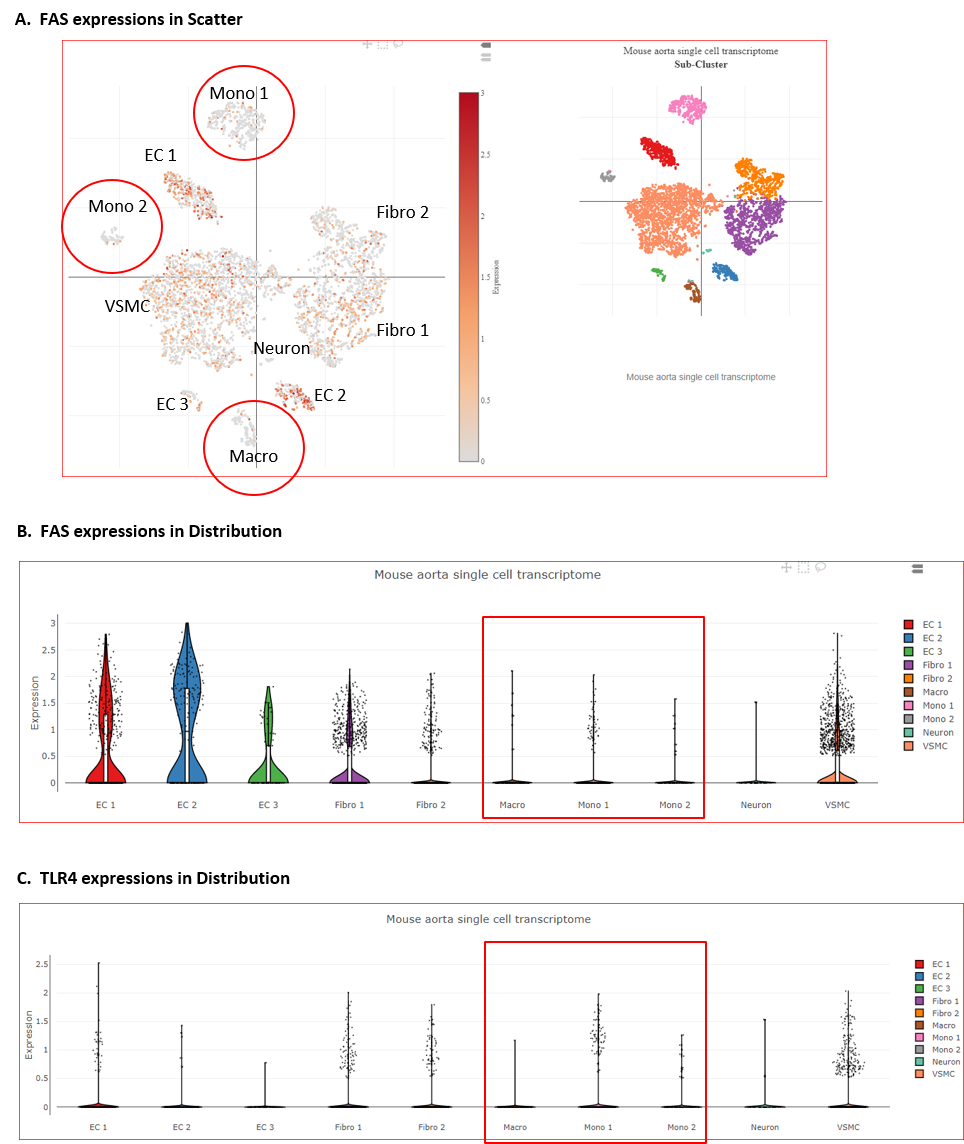

Supplement: Supplementary Figure 6 — Fas mRNA transcripts are found in mouse aortic monocyte 1, monocyte 2 and macrophages. The data mining analyses were performed on the Single Cell RNA-Seq database of the Broad Institute of MIT and Harvard (Single CellBeta Portal; https://singlecell.broadinstitute.org/single_cell/study/SCP289/single-cell-analysis-of-the-normal-mouse-aorta-reveals-functionally-distinct-endothelial-cell-populations#study-summary, PMID: 31146585). (A) Fas expressions in monocytes and macrophages were circled in red in the Scatter; (B) Fas expressions in monocytes and macrophages were boxed in red in the Distribution; (C) Toll-like receptor 4 (TLR4) expressions in monocytes and macrophages were also boxed in read in the Distribution. [file Image_6.tif]

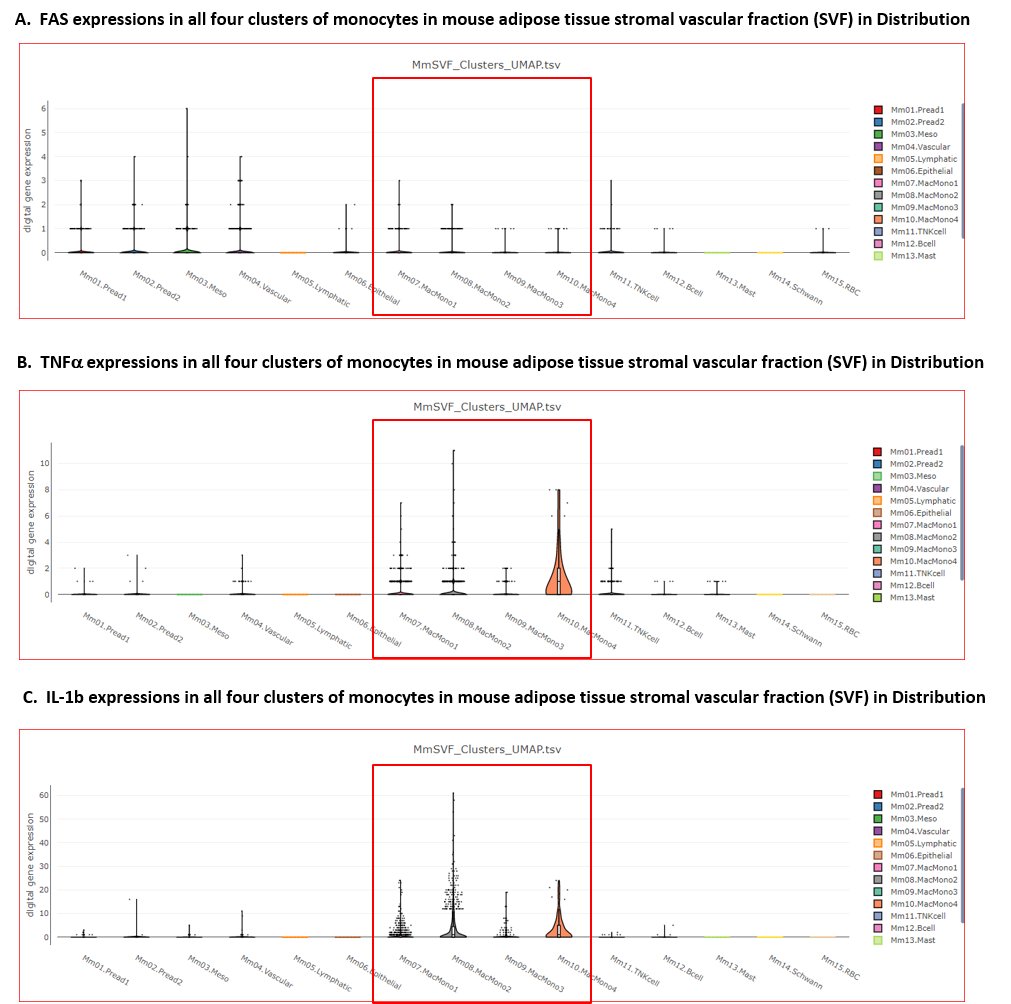

Supplement: Supplementary Figure 7 — The expressions of Fas, TNFα, and IL-1b are found in all the four clusters of monocytes/macrophages in mouse adipose tissue stromal vascular fraction (SVF). The data mining analyses were performed on the Single Cell RNA-Seq database of the Broad Institute of MIT and Harvard (Single CellBeta Portal; https://singlecell.broadinstitute.org/single_cell/study/SCP708/mouse-adipose-stromal-vascular-fraction#study-summary). (A) Fas expressions in monocytes and macrophages were boxed in red in the Scatter; (B) TNFα expressions in monocytes and macrophages were boxed in red in the Distribution; (C) IL-1b expressions in monocytes and macrophages were also boxed in read in the Distribution. [file Image_7.tif]

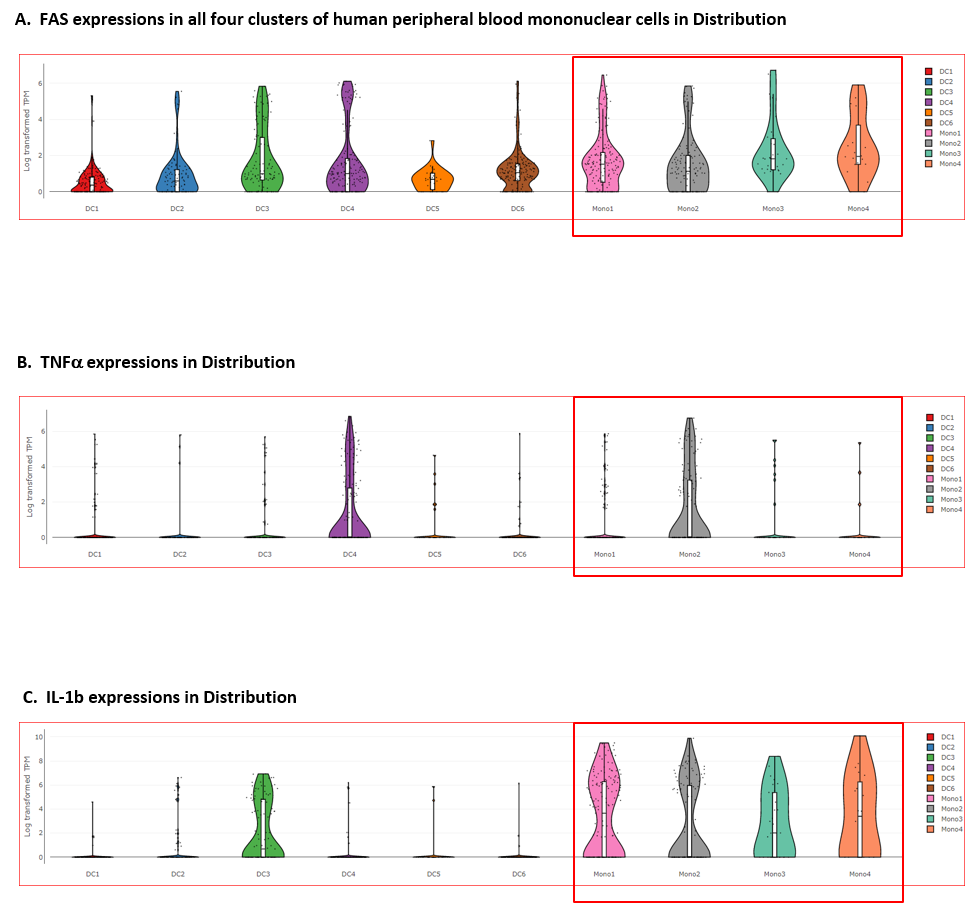

Supplement: Supplementary Figure 8 — The expressions of Fas, TNFα, and IL-1b are found in all the four clusters of monocytes/macrophages in human blood. The data mining analyses were performed on the Single Cell RNA-Seq database of the Broad Institute of MIT and Harvard (Single CellBeta Portal; https://singlecell.broadinstitute.org/single_cell/study/SCP43/atlas-of-human-blood-dendritic-cells-and-monocytes#study-summary; PMID: 28428369). (A) Fas expressions in monocytes and macrophages were boxed in red in the Scatter; (B) TNFα expressions in monocytes and macrophages were boxed in red in the Distribution; (C) IL-1b expressions in monocytes and macrophages were also boxed in read in the Distribution. [file Image_8.tif]

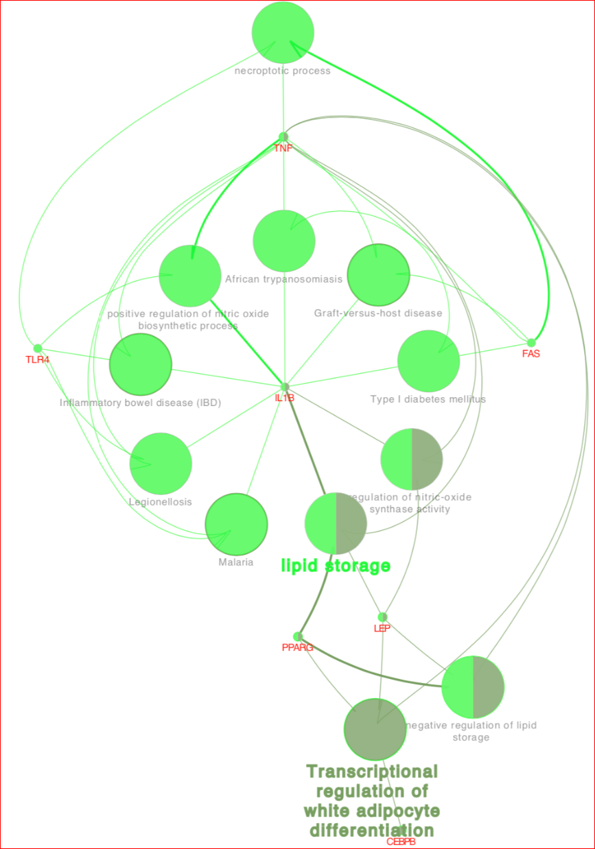

Supplement: Supplementary Figure 9 — Extended high fat diet (HF) feeding-upregulated proinflammatory regulators including Fas (CD95), TNFα, IL-1b, and TLR4 overcome miR155 deficiency and promote resurgence of atherosclerosis. We reported that the expressions of C/EBP, PPARg, leptin (Lep), and resistin are upregulated in HF-fed MHO mice (PMIDs: 27856635; 30369883); and we also found that the expressions of CD95, TNFα, IL-1b and TLR4 are increased in HF-fed MHO mice in this study. The Cytoscape analyses (https://cytoscape.org/; the network data integration, analysis and visualization database) indicate that pro-inflammatory and pro-immune, lipid storage and white adipose tissue differentiation pathways are interplayed and promote resurgence of atherosclerosis and MHO transition to MUO. [file Image_9.tif]
