# Supplementary material for: A Novel Subset of CD95+ Pro-Inflammatory Macrophages Overcome miR155 Deficiency and May Serve as a Switch From Metabolically Healthy Obesity to Metabolically Unhealthy Obesity
Source: Front Immunol. 2021 Jan 7;11:619951. doi: 10.3389/fimmu.2020.619951 (PMC7817616; doi:10.3389/fimmu.2020.619951)
Supplement: Supplementary file 10 [file Presentation_1.pptx]

## Slide 1
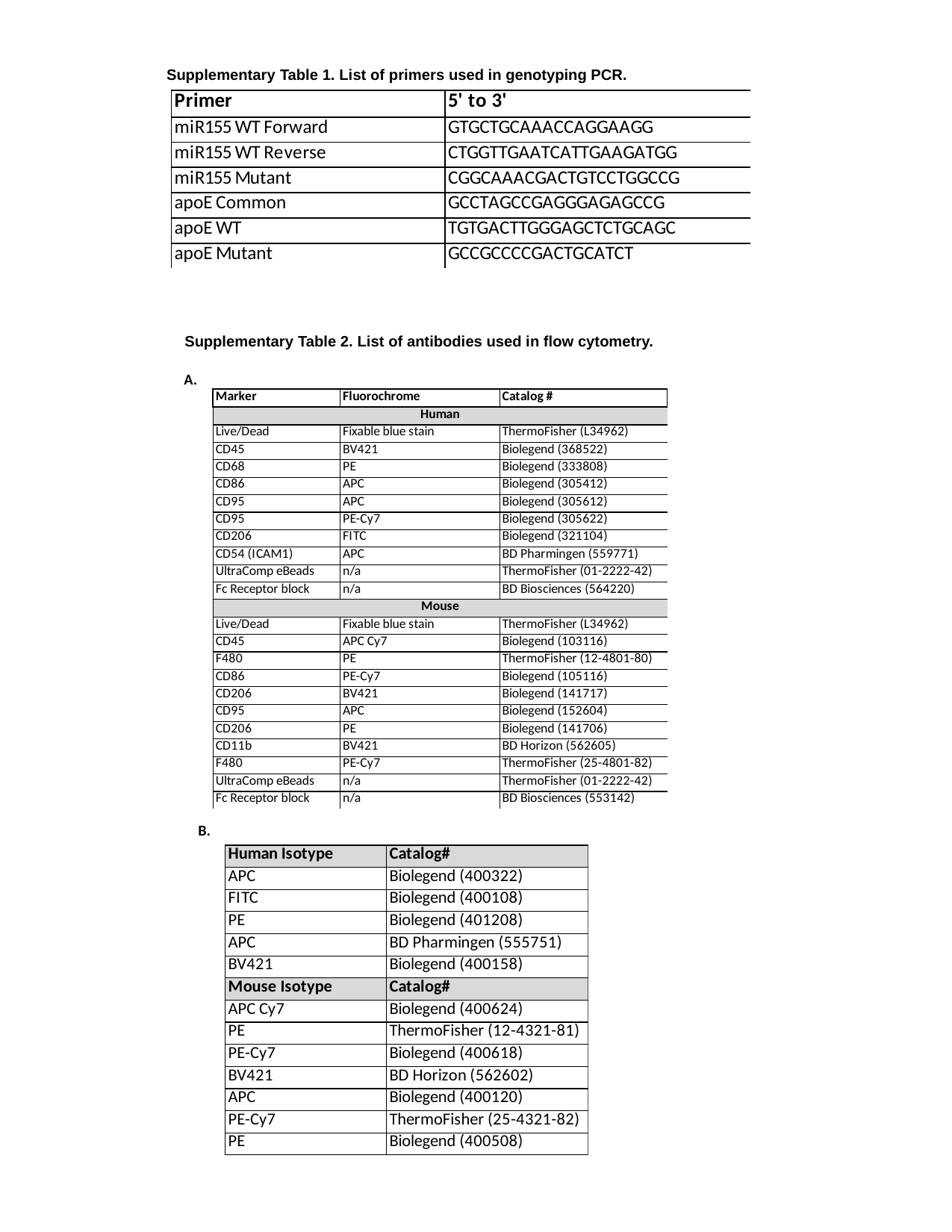

Supplementary Table 1. List of primers used in genotyping PCR.
Supplementary Table 2. List of antibodies used in flow cytometry.
A.
B.

## Slide 2
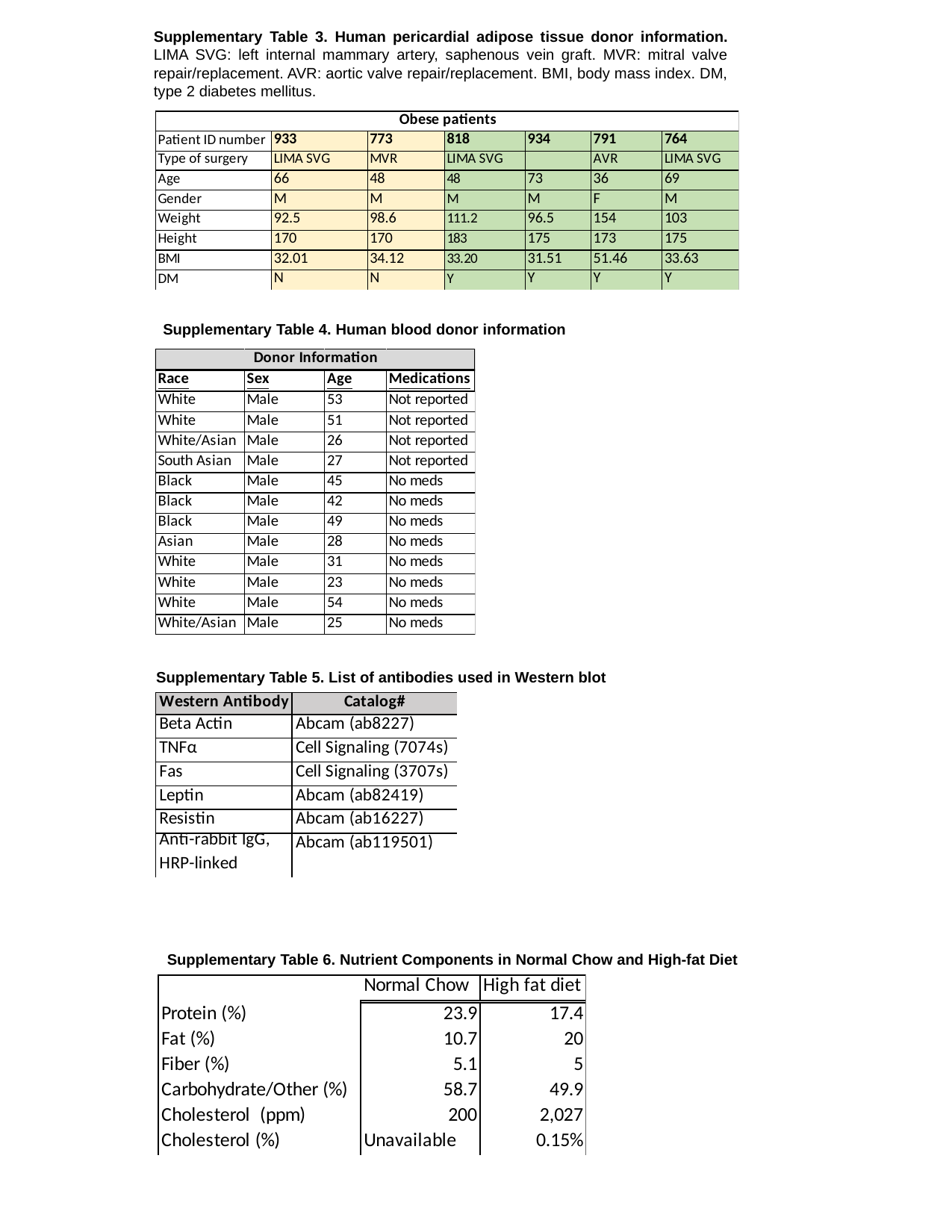

Supplementary Table 3. Human pericardial adipose tissue donor information. LIMA SVG: left internal mammary artery, saphenous vein graft. MVR: mitral valve repair/replacement. AVR: aortic valve repair/replacement. BMI, body mass index. DM, type 2 diabetes mellitus.
Supplementary Table 4. Human blood donor information
Supplementary Table 5. List of antibodies used in Western blot
Supplementary Table 6. Nutrient Components in Normal Chow and High-fat Diet
